# Supplementary material for: Modeling geographic vaccination strategies for COVID-19 in Norway
Source: PLoS Comput Biol. 2024 Jan 31;20(1):e1011426. doi: 10.1371/journal.pcbi.1011426 (PMC10861074; doi:10.1371/journal.pcbi.1011426)
Supplement: S1 Text — This includes more detailed descriptions of the models and additional results. (PDF) [file pcbi.1011426.s001.pdf]

Modeling geographic vaccination strategies for COVID-19 in Norway
S1 Text: The supplementary document

Louis Yat Hin Chan[1*], Gunnar Rø[1], Jørgen Eriksson Midtbø[1], Francesco Di Ruscio[1], Sara Sofie Viksmoen Watle[2], Lene Kristine Juvet[2], Jasper Littmann[3,5], Preben Aavitsland[3,6], Karin Maria Nygård[4], Are Stuwitz Berg[2], Geir Bukholm[3,7], Anja Bråthen Kristoffersen[1], Kenth Engø-Monsen[8], Solveig Engebretsen[9], David Swanson[10], Alfonso Diz-Lois Palomares[1], Jonas Christoffer Lindstrøm[1], Arnoldo Frigessi[11], Birgitte Freiesleben de Blasio[1,11]

1 Department of Method Development and Analytics, Norwegian Institute of Public Health, Oslo, Norway
2 Department of Infection Control and Vaccines, Norwegian Institute of Public Health, Oslo, Norway
3 Division of Infection Control, Norwegian Institute of Public Health, Oslo, Norway
4 Department of Infectious Diseases and Preparedness, Norwegian Institute of Public Health, Oslo, Norway
5 Bergen Centre for Ethics and Priority Setting (BCEPS), University of Bergen, Bergen, Norway
6 Pandemic Centre, University of Bergen, Bergen, Norway
7 Faculty of Chemistry, Biotechnology and Food Sciences, Norwegian University of Life Sciences, Ås, Norway
8 Smart Innovation Norway, Halden, Norway
9 SAMBA, Norwegian Computing Center, Oslo, Norway
10 Department of Biostatistics, MD Anderson Cancer Center, University of Texas, Texas, United States of America
11 Oslo Centre for Biostatistics and Epidemiology, University of Oslo and Oslo University Hospital, Oslo, Norway

* imlouischan@gmail.com

S1 Model descriptions

Here we give more detailed descriptions of the two models: the individual-based model (IBM) is a highly detailed model while the meta-population model (MPM) uses a population-based structure to describe the spread.

S1.1 Individual-based model (IBM)

In this study, we adapt a stochastic IBM previously used for studying Methicillin-resistant *Staphylococcus aureus* (MRSA) transmission [1] to model the spread of COVID-19. Our model incorporates individual-level factors such as age, location, occupation, risk status, epidemiological status, hospitalization status, and vaccination status. We characterize our synthetic population using Norwegian census data, and assign individuals to households based on population density and age distribution in 13,521 contiguous areas known as Grunnkrets in Norwegian or Basic circuits in English [2].

Occupation locations, including schools, universities, workplaces, and those who are unemployed or retired, are assigned based on age. The simulated age-specific contact matrices in different routes are depicted in Fig A. Our model aligns with other similar models used to study different interventions against COVID-19 [3–8].

S1.1.1 Households

To generate a distribution of household sizes for individuals, we utilize a two-dimensional size-age distribution, as illustrated in Fig BA, from Statistics Norway [10]. Specifically, individuals are assigned to households ranging in size from 1 (i.e., single living) to a maximum of 7 individuals, with households determined based on the size-age distribution. Individuals aged 15 years and younger are assigned to households with at least two individuals. Individuals aged 88 years and older are aggregated into a single category, as data availability indicates that they are primarily living alone. The methodology underlying household construction follows the algorithm used in [1, 9].

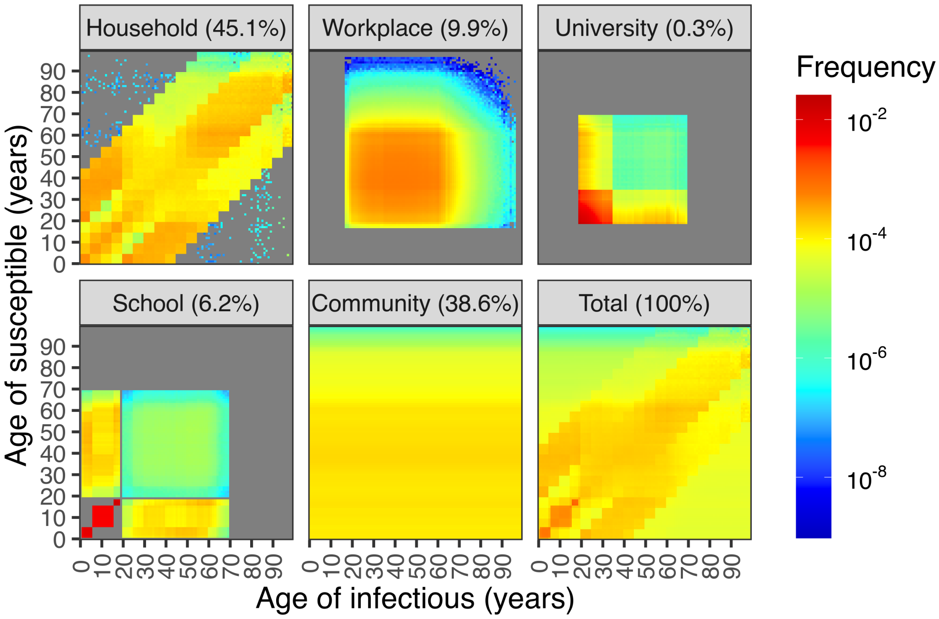

Fig A. The age-specific contact pattern in the IBM. The age-specific contact frequencies of each transmission setting and total sum across all settings from low to high are represented by a color gradient from blue to red. The total frequency is obtained by the linear combination of each transmission setting, with the corresponding contributing proportion shown in brackets. These contact patterns are similar to those in the modeling study in the UK [9].

S1.1.2 Occupations

Individuals are assigned in schools, universities or workplaces according to their ages and specific places according to size distributions of occupations. Specifically, children between 1 and 5 years old attend kindergarten, those between 6 and 15 years old attend primary school, those between 16 and 18 years old attend secondary school, and those over 19 years old attend university. Individuals who are 17 years old or older and not attending university or unemployed are assigned to workplaces [1].

In the context of the COVID-19 pandemic, it is assumed that 20% of employees work from home as a result of Norwegian interventions implemented in 2021. In addition, some individuals are specifically assigned to work as healthcare workers based on an age distribution, and they are given priority for vaccination within the population.

S1.1.3 Community

In the individual-based model, contact between individuals can occur through community activities as long as they are not completely isolated from society. Community contacts refer to interactions that take place outside of the home, school, or workplace, and may include activities such as taking public transportation, grocery shopping, dining out, socializing with friends, and visiting non-cohabiting family members. Age-specific contact data were collected in Norway in 2017 [11] and show that younger individuals have higher rates of community contact than older individuals.

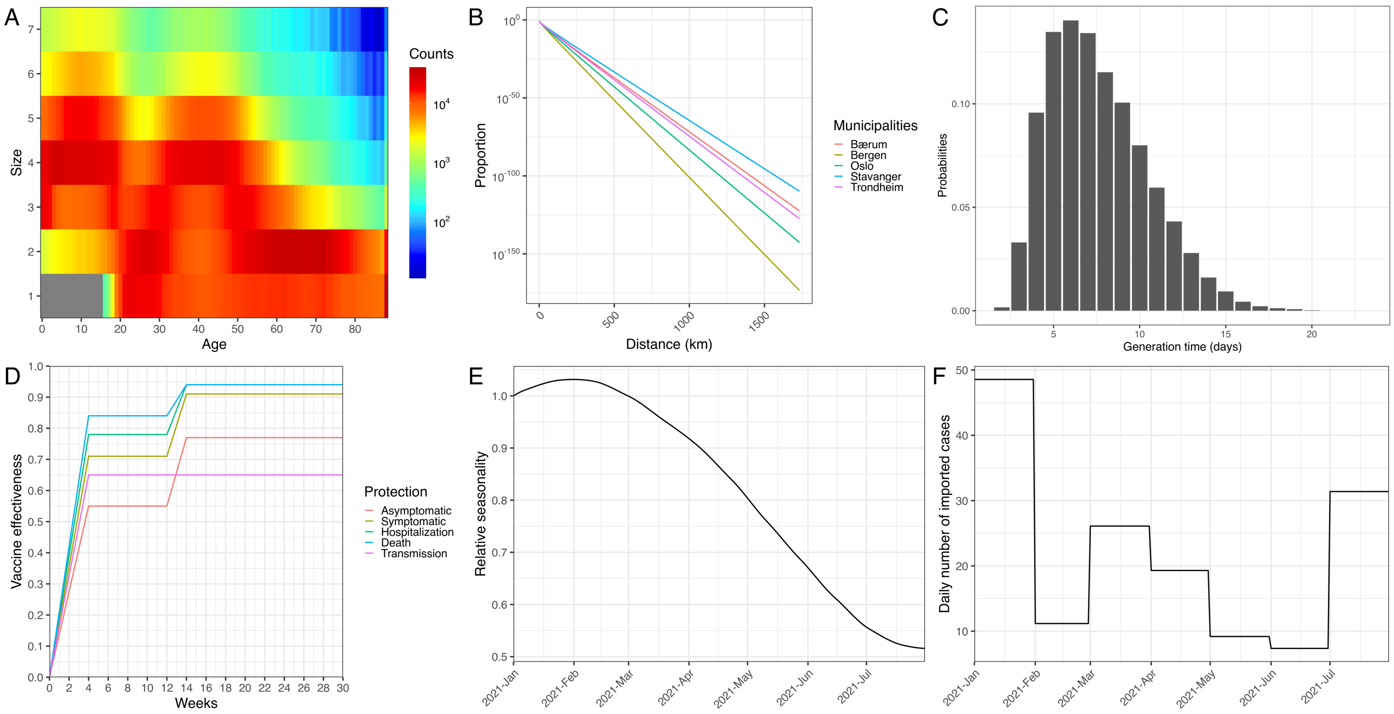

Fig B. The model assumptions. A: Age-specific distribution of household sizes in Norway. The gray color represents that those 15 years old and younger are not single living. The rightmost column shows the group of individuals 88 years old or older, who are primarily living alone. Household counts are shown on a log-scale. B: The municipality-specific mobility pattern in Norway. The proportions of radius of gyration of the top 5 most populated municipalities (Oslo, Bergen, Trondheim, Stavanger, Bærum) are shown in a log-scale. C: The generation time distribution. Probability mass function (PMF) of the generation time distribution of the individual-based model is illustrated using results from one simulation. The median generation time is 7 days. D: The growth of vaccine effectiveness. The effectiveness of the first dose increases linearly from zero to the first full effect in 28 days (4 weeks) and then remains constant for 56 days (8 weeks) after reaching the first full effect. The time interval between first and second doses is assumed to be 84 days (12 weeks). The effectiveness of the second dose increases linearly to the second full effect in 14 days (2 weeks) after vaccination. E: The seasonality of transmission rate. The relative seasonality of transmissibility starts from 1.0 on the 1st of January and varies according yearly temperature data in Norway. The 50% seasonality refers to a 50% decrease in the lowest transmissibility during summer compared to the highest in winter. F: The importation of cases. The daily imported cases are average values based on monthly national data from MSIS. The observed monthly cases from January to July 2021 were 1505, 313, 809, 579, 285, 221, 973. These cases were then distributed among different counties and age groups according to empirical distributions.

Additionally, we account for individual mobility, where contact patterns can vary by distance. We obtained mobility data for each municipality in Norway from Telenor in 2021 [12]. These data reveal that long-range movements, such as travel from Oslo to Bergen, occur less frequently than short-range movements, such as travel between different districts in Oslo. Fig BB shows the fitted distribution of mobility distances based on the proportion of radius of gyration for the top five most populated municipalities in Norway (Oslo, Bergen, Trondheim, Stavanger, Bærum).

S1.1.4 Epidemiological status

Each individual in the model is assigned an epidemiological status according to a SEIR-like epidemiological model, which is illustrated in gray in Fig C. A susceptible individual can become infected by coming into contact with asymptomatic, pre-symptomatic, or symptomatic infectious individuals. Once infected, the individual becomes exposed, but is not yet infectious, and may develop either asymptomatic or symptomatic illness in the next few days. The probability of remaining asymptomatic is 40%, while the probability of developing pre-symptomatic illness before becoming symptomatic is 60%. This is the assumption throughout our analyses during the pandemic, and the 40% asymptomatic fraction aligns with findings from a systematic review [13]. Symptomatic individuals may either recover or die, as shown in red and blue in Fig C, respectively. Asymptomatic individuals who become infected are assumed to recover without developing symptoms.

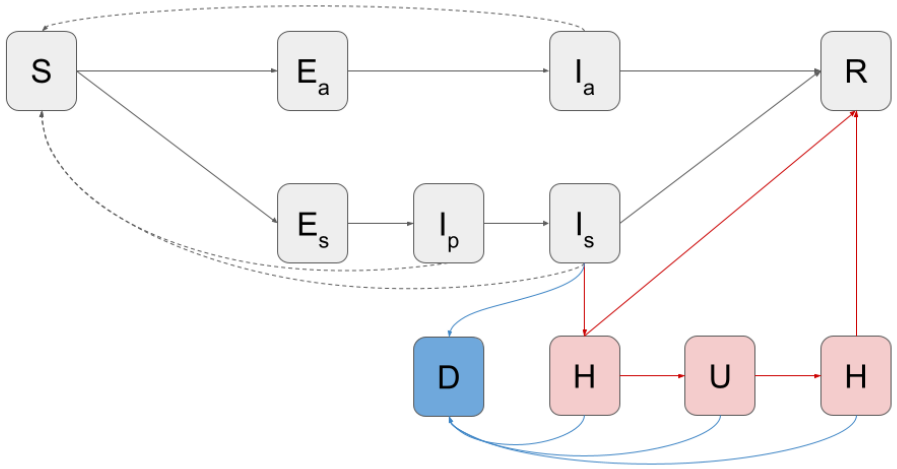

Fig C. The epidemiological and hospitalization model. Susceptible individuals (S) who have not been exposed to the virus or the vaccine, upon exposure, enter a latent state (E_a or E_s) before becoming infectious. Symptomatic individuals enter a pre-symptomatic state (I_p) before developing symptoms (I_s). Asymptomatic infections (I_a) are assumed to recover (R) without developing symptoms. Those with symptomatic infections (I_s) may recover (R) or require hospitalization (H) due to severe illness. Hospitalization can lead to recovery (R), or admission to the ICU (U) followed by a second stay in the hospital ward (H), and eventual recovery (R). Deaths (D) occur from the symptomatic state or during hospitalization. The epidemiological statuses are shown in gray. S: susceptible. E_a: asymptomatic exposed. I_a: asymptomatic infectious. E_s: symptomatic exposed. I_p: pre-symptomatic infectious. I_s: symptomatic infectious. R: recovered. The solid directed lines are transitions between statuses and the dashed directed lines represent infectious transmissions. The symptomatic infectious people may be hospitalized shown in red statuses and/or die shown in blue as extra layers. H: hospitalized. U: ICU admission. D: died.

The time spent of each status following exposure is represented by a gamma distribution and is age-independent. Table A shows the parameters for each status. The median generation time, defined as the time between the infection in a primary case and a secondary case, is approximately 7 days. An example of the generation time distribution from one simulation is shown in Fig BC.

The probability of death given a symptomatic infection is age-specific and depends on whether an individual belongs to a risk group, i.e. risk status. These values are calibrated using Norwegian registry data, which is explained in the

following section.

Table A. The time spent and infectiousness of compartments in the transmission model.

Compartments	Mean (days)	Shape	Scale	Infectiousness
Presymptomatic latent (Es)	3.0	7.5	0.4	0
Asymptomatic latent (Ea)	3.0	7.5	0.4	0
Presymptomatic (Ip)	2.0	5.0	0.4	1.25
Symptomatic (Is)	4.8	6.0	0.8	1.00
Asymptomatic (Ia)	4.8	6.0	0.8	0.10

Individuals who are exposed to the virus enter a latent period during which they are not infectious. After this period, 40% of them may become asymptomatic, which means they are less infectious than those who develop symptoms. The table provides information about the gamma distribution of the time spent in each status, including the shape and scale parameters, as well as the corresponding mean values.

S1.1.5 Hospitalization status

We account for the possibility that individuals with symptomatic infections may develop severe illness and require hospitalization or ICU admission. While some hospitalized individuals may not require ICU admission and can recover and be discharged after a few days, others may need to stay in the hospital longer, especially those who require ICU admission. Age is the only factor that influences the time spent in hospital or ICU.

The time spent in each status, including the time from symptom onset to hospitalization, follows negative binomial distributions with their probabilities and size parameters. We first model the time from symptom onset to hospitalization and then split it into two ways to model individuals requiring ICU admission and those who do not. For those who do not require ICU admission, the length of stay in the hospital is captured by one distribution. For those who require ICU admission, we model the time from hospitalization to ICU admission, length of stay in ICU, and length of stay in the hospital after ICU admission in three separate steps.

The probabilities of hospitalization given symptomatic infection are age-specific and depend on whether individuals belong to a risk group. These probabilities are calibrated using Norwegian registry data, which we present in the next section.

S1.1.6 Vaccination status

Each individual can be vaccinated on a specific date. However, after vaccination, it takes some time for immunity to develop, and protection increases gradually over the following weeks. In this study, we assume a leaky type of vaccine effectiveness instead of an all-or-nothing effect [14]. This means that while all vaccinated people are not fully protected, their risk of infection is reduced by a certain fraction in each contact. Additionally, we assume no waning immunity during the simulation period due to its relatively short duration.

Our assumptions for vaccine effectiveness are as follows. The protection probability increases linearly from zero to the first full effect during the first 28 days (4 weeks) after vaccination. The effectiveness then remains constant for the following 56 days (8 weeks), as the time interval between the first and second doses is assumed to be 84 days (12 weeks). We assume that everyone who takes the first dose will take the second, and that the effectiveness of the second dose increases linearly to the second full effect over the following 14 days (2 weeks).

The vaccine provides several protections against (i) symptomatic infection, (ii) asymptomatic infection, (iii) hospitalization, (iv) death and (v) transmissibility. The full protection are (i) 71/91%, (ii) 55/77%, (iii) 78/94%, (iv)

84/94% and (v) 65/65% after the first/second dose, respectively. The values are based on the recommendation reports by the Norwegian Institute of Public Health (NIPH) [15–18] and are similar to estimates from another study [19]. Table B and Fig BD show the vaccine effectiveness and its growth over time.

The conditional effectiveness against severe illness (i.e. hospitalization or death) given symptomatic infection can be calculated by Eq (S1).

$$\mathrm{VE}_{\mathrm{H|I}} = 1 - \frac{1 - \mathrm{VE}_{\mathrm{H}}}{1 - \mathrm{VE}_{\mathrm{I}}}, \tag{S1}$$

where $\mathrm{VE}_{\mathrm{H|I}}$, VE_{H}, VE_{I} are the vaccine effectiveness against severe illness given symptomatic infection, severe illness and symptomatic infection, respectively. For example assuming vaccine reaches the maximum effectiveness (i.e. more than 2 weeks after the second dose), we have $\mathrm{VE}_{\mathrm{I}} = 0.91$, $\mathrm{VE}_{\mathrm{H}} = 0.94$ and $\mathrm{VE}_{\mathrm{H|I}} = 0.33$, which means that the risk of severe illness given symptomatic infection is reduced by 33%.

Table B. The vaccine effectiveness against different health statuses.

**Vaccine effectiveness against**	**1st dose (%)**	**2nd dose (%)**
Symptomatic	71	91
Asymptomatic	55	77
Hospitalization	78	94
Death	84	94
Transmissibility	65	65

The effectiveness of the first dose increases linearly from zero to the first full effect in 28 days (4 weeks) and then remains constant for 56 days (8 weeks) as the time interval between first and second doses is assumed to be 84 days (12 weeks). The effectiveness of the second dose increases linearly to the second full effect in 14 days (2 weeks) after vaccination.

S1.1.7 Seasonality

Apart from control interventions, the transmission rate is known to vary seasonally, with higher rates occurring in the winter and lower rates in the summer. We assume a 50% relative difference between the highest and lowest seasonal rates, which is defined based on the temperature over a year. Fig BE illustrates the seasonal variation in the transmission rate from January to July.

S1.1.8 Importation

Importation of infectious cases is simulated by converting susceptible individuals to either symptomatic or asymptomatic infected individuals. We select individuals based on age- and location-specific empirical distributions derived from observed data. At each time step (each day) in the simulation, a daily importation number is drawn from a Poisson distribution with a time-varying mean, which is based on the data presented in Fig BF.

S1.1.9 Calibration

We calibrated the ratios between the four setting-specific betas (household, school, workplace, and community) using COVID-19 test positive data. The main contributions to the positive cases were infections within households and in the community, which includes all routes other than households, schools, or workplaces.

To account for time-specific changes, we divided the seven-month simulation period into three intervals, separated by two change points on the 28th of January and 11th of March 2021. We selected the change points based on the local

minimum and maximum rolling averages of daily hospital admissions and shifted them back 12 days to account for the average delay between infection and hospitalization.

To calibrate the IBM, we used 12 free parameters, including beta (i.e. transmission rates) during three time periods and susceptibilities of nine age groups to capture time-specific changes and age-specific differences, respectively. We sampled 100,000 parameters using the Latin hypercube sampling (LHS) approach and selected the best 10 sets based on the least squares method. The least squares error was calculated using a data set consisting of two marginal distributions: $N_t = 212$ time points of daily hospital admissions and the seven-month cumulative admissions of $N_a = 9$ age groups. The least squares error is

$$\mathrm{L}^2 = \sum_{t=1}^{N_t} \left(\sum_{a=1}^{N_a} x_{t,a} - \sum_{a=1}^{N_a} y_{t,a} \right)^2 + \sum_{a=1}^{N_a} \left(\sum_{t=1}^{N_t} x_{t,a} - \sum_{t=1}^{N_t} y_{t,a} \right)^2 , \tag{S2}$$

where $x_{t,a}$ and $y_{t,a}$ are the daily hospital admission of model output and observed data at time t and of age group a, respectively. The marginals $\sum_{a=1}^{N_a} y_{t,a}$ and $\sum_{t=1}^{N_t} y_{t,a}$ are the daily hospital admissions of all ages at time t and the 7-month total hospital admissions of each age group a, respectively.

To narrow down the range of parameters ranging from 0 to 1, we performed several rounds of Latin hypercube sampling (LHS). Fig D shows the final round parameter distributions, with higher beta values in February corresponding to the growth of cases in March.

Our estimated susceptibilities to infection showed a decrease with age, except for the 50-59 age group. This is in contrast to findings in other modeling studies [20–22] and cohort studies [23, 24]. Several factors could explain these differences. First, we applied age-specific risk ratios of the Alpha variant in Norway [25], which had large uncertainties and may have overestimated values for younger age groups or underestimated values for older age groups. Therefore, susceptibilities for older individuals may have been underestimated. Second, our age-specific contact patterns were taken from 2017 before the pandemic, while contact matrices in other modeling studies [21, 22] were separated into pre-pandemic and post-lockdown periods. It is possible that older individuals reduced their contacts more than younger individuals during the pandemic, leading to underestimated susceptibilities for older age groups. Third, vaccine effectiveness may have been lower for older individuals [26], while we assumed equal effectiveness across all age groups, which may have overvalued vaccine effectiveness for older individuals and led to underestimated susceptibilities for this group. The national recommendation reports by NIPH addressed this issue by splitting vaccine effectiveness into two categories: those aged 65 years and younger and those older than 65 years [27–30].

Fig E shows the fits of the model to the data. The estimated numbers of infections are higher than the reported cases due to under-detection, particularly in younger age groups. The calibration process uses only daily and age-specific hospital admissions to estimate the 12 free parameters. The good match between the observed and modeled ICU admissions and deaths in Fig EA validates the calibration method. Fig EC, which shows the geographic-specific differences not used in the estimation, also supports the model accuracy.

Using the observed number of deaths and the estimated mean number of infections, we calculate the age-specific probabilities of death given infection. The age-specific probabilities of ICU admission given hospitalization are calculated based on the observed numbers of ICU admissions and hospitalizations. The resulting age-specific probabilities of death and ICU admission are presented in Table C. Note that these probabilities are calculated in the absence of vaccination and that the vaccine effectiveness on the probabilities depends on the timing of vaccination for each individual.

To assess the sensitivity of the results to the choice of parameters, we also calibrate the model using the probabilities of hospitalization without the factor of 0.5. In this case, the estimated number of infections is roughly halved, and the resulting probabilities of death given infection are approximately doubled.

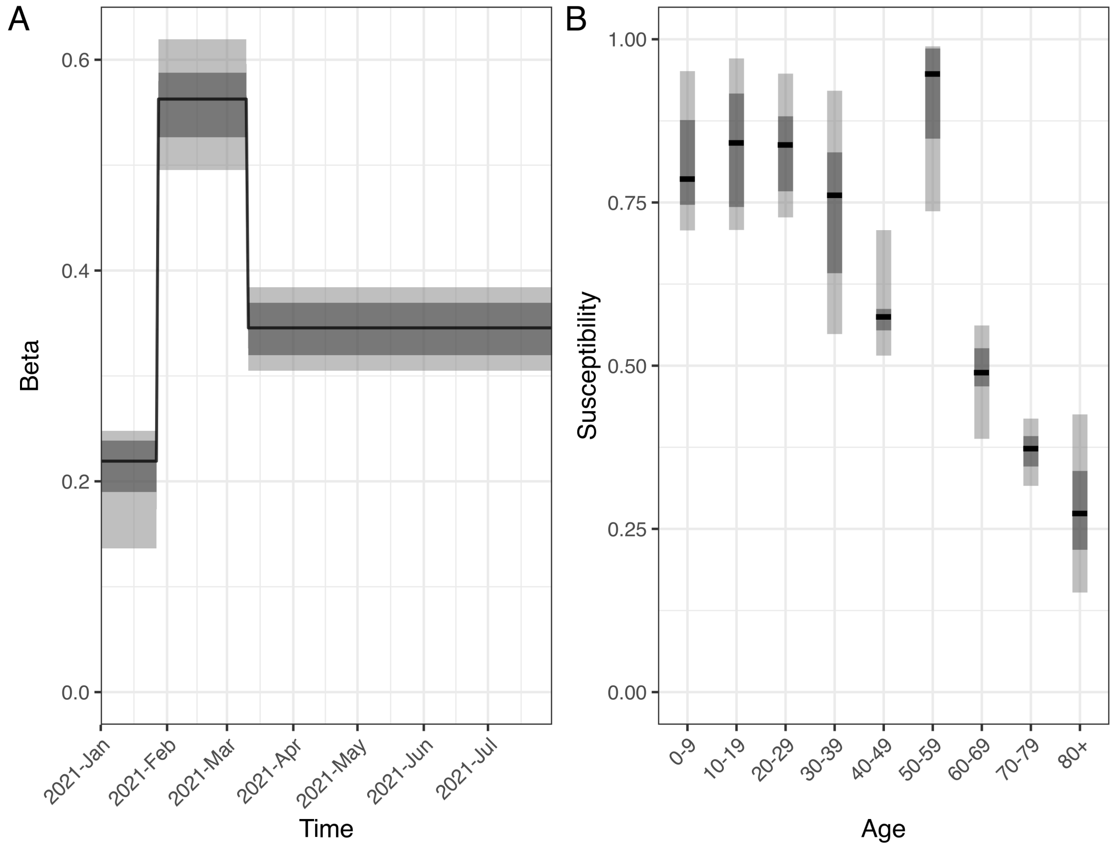

Fig D. The marginal distributions of 12 estimated parameters in the IBM. (A) Beta in 3 time periods and (B) susceptibilities of 9 age groups are sampled from Latin hypercube sampling (LHS) approach. The black lines show the median values, and the darker (lighter) gray areas show the ranges of 25th and 75th percentiles, i.e. lower and upper quantiles (2.5th and 97.5th percentiles) of the estimated parameters.

S1.2 Meta-population model (MPM)

The meta-population model (MPM) is based on an epidemiological model identical to the one used in the individual-based model (IBM). The model incorporates meta-populations that are characterized by age, risk factor, and geography. The geographic regions are based on the five regions of Norway (North, West, Mid, East, and South) and different prioritization schemes. This results in a representation of every possible combination of prioritization (*Plus*, *Neutral*, or *Minus*) and region. In the baseline model, we have 10 geographic regions, 9 age groups, and 2 risk profiles, resulting in 180 separate meta-populations. Vaccination is incorporated as an additional layer of meta-populations, resulting in 540 meta-populations in the baseline when considering 3 vaccination states (No vaccine, first dose, and second dose). The vaccinated groups exhibit different values for susceptibility, severity, and transmissibility compared to the non-vaccinated groups.

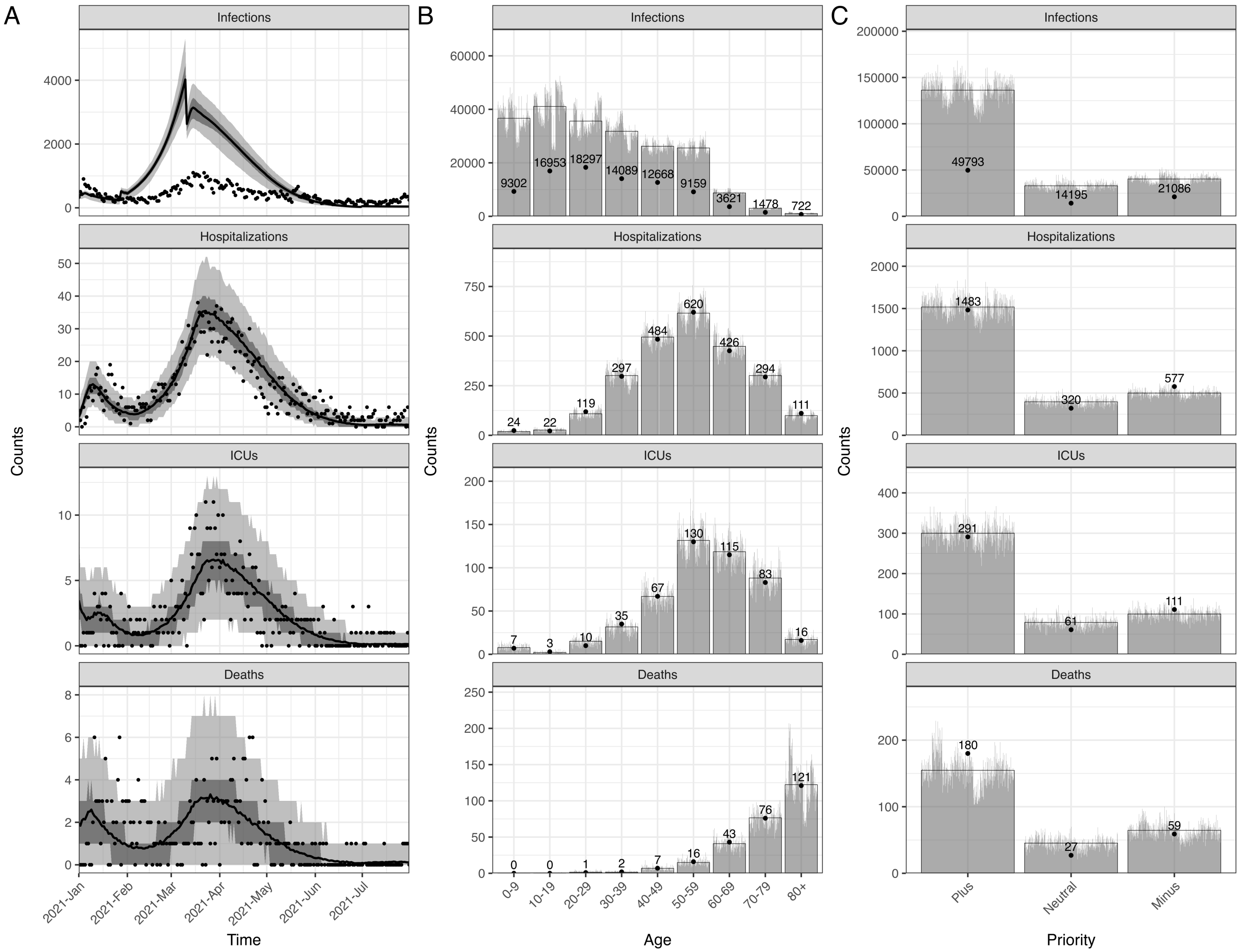

Fig E. The calibrated IBM and data. A: The time series data of all ages. The lines show the model fits with their 50 and 95% prediction intervals represented by gray areas. The dots show the observed data. B: The age distribution of total counts. C: The geographic-specific priority distribution of total counts. The gray bars show the counts of each simulation and the full bars with black borders show the mean of all simulations. The data are shown in dots with their exact numbers.

The model is defined by the following equations (see Table D for description of variables):

$$S^i(t+\delta t) = S^i(t) - X_1^i(t) - \text{import}^i(t) \tag{S3}$$
$$Ea^i(t+\delta t) = Ea^i(t) + X_2^i(t) - X_3^i(t) \tag{S4}$$
$$Es^i(t+\delta t) = Es^i(t) + X_4^i(t) - X_5^i(t) \tag{S5}$$
$$P^i(t+\delta t) = P^i(t) + X_5^i(t) - X_6^i(t) \tag{S6}$$
$$A^i(t+\delta t) = A^i(t) + X_3^i(t) - X_7^i(t) \tag{S7}$$
$$I^i(t+\delta t) = I^i(t) + X_6^i(t) - X_8^i(t) + \text{import}^i(t) \tag{S8}$$
$$H^i(t+\delta t) = H^i(t) + X_9^i(t) - X_10^i \tag{S9}$$
$$ICU_1^i(t+\delta t) = ICU_1^i(t) + X_{11}^i(t) - X_{12}^i(t) \tag{S10}$$
$$ICU_2^i(t+\delta t) = ICU_2^i(t) + X_{12}^i(t) - X_{13}^i(t) \tag{S11}$$
$$ICU_3^i(t+\delta t) = ICU_3^i(t) + X_{13}^i(t) - X_{14}^i(t) \tag{S12}$$
$$R^i(t+\delta t) = R^i(t) + X_{15}^i + X_{16}^i + X_{17}^i + X_{18}^i \tag{S13}$$
$$D^i(t+\delta t) = D^i(t) + X_{19}^i(t) + X_{20}^i(t) + X_{21}^i(t) + X_{22}^i(t) \tag{S14}$$

Table C. The age-specific probabilities of death given infection and ICU admission given hospitalization in the IBM.

	Probabilities of death (%)		Probabilities of ICU admission (%)
**Age groups**	**Without risk factors**	**With risk factors**	**All population**
0-9	0.00009	0.0002	33.3
10-19	0.0004	0.0007	6.9
20-29	0.007	0.01	12.2
30-39	0.008	0.01	9.9
40-49	0.04	0.07	13.1
50-59	0.08	0.1	20.8
60-69	0.6	1.06	25.5
70-79	2.9	5.4	28.5
80+	13.0	23.9	17.1

We assume that the probabilities of death depend on both age and risk status while the probabilities of ICU admission depend on age only. Based on the observed number of deaths and the estimated mean number of infections, we calculate the age-specific probabilities of death given infection. Similarly, based on the observed numbers of ICU admissions and hospitalizations, we calculate the age-specific probabilities of ICU admission given hospitalization.

The infection process, given by X_1^i is simulated from a binomial distribution with a probability given by the force of infection, Λ_i

$$X_1^i(t) \quad \sim \quad \text{Binom}(S^i(t), 1 - \exp(-\Lambda^i(t) * \delta t)). \tag{S15}$$

The force of infection is

$$\Lambda_i(t) = \beta_i sus_i M_{ij} trans_j (inf_p P^j(t) + inf_s I^j(t) + inf_a A^j(t)), \tag{S16}$$

where $sus_i = f_a^i sus_a^i + f_s^i * sus^i$. The contact matrix M_{ij} is the outer product of an age-based contact matrix based on a survey in Norway and regional contact matrix. We assume the people in risk groups have the same contact pattern as the people not in risk groups. Fig F shows the age-based contact matrix.

The other transition probabilities X_i are determined by the appropriate probabilities and lengths of stay in various

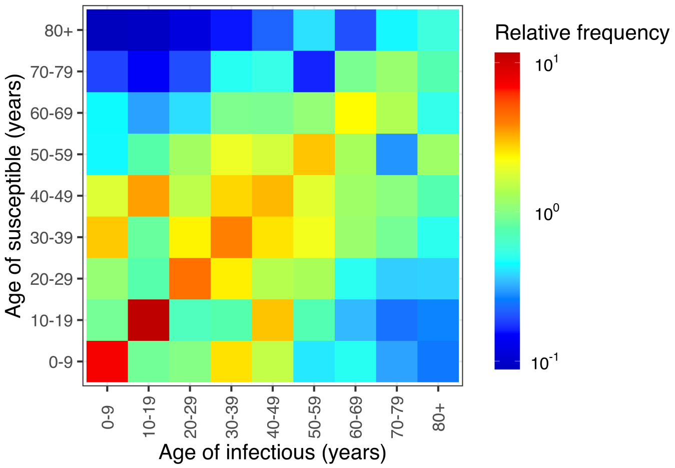

Fig F. The age-specific contact pattern in the MPM. The age-specific relative contact frequencies based on a survey in Norway [11] from low to high in log10-scale are represented by color from blue to red.

compartments.

$$
\begin{aligned}
X_2^i(t) &\sim \text{binom}(X_1^i(t), f_a^i) && \text{(S17)}\\
X_3^i(t) &\sim \text{binom}(Ea^i(t), 1 - exp(-\delta t/D_E) && \text{(S18)}\\
X_4^i(t) &= X_1^i(t) - X_2(t)^i && \text{(S19)}\\
X_5^i(t) &\sim \text{binom}(Es^i(t), 1 - exp(-\delta t/D_E) && \text{(S20)}\\
X_6^i(t) &\sim \text{binom}(P^i(t), 1 - exp(-\delta t/D_P) && \text{(S21)}\\
X_7^i(t) &\sim \text{binom}(A^i(t), 1 - exp(-\delta t/D_A) && \text{(S22)}\\
X_8^i(t) &\sim \text{binom}(I^i(t), 1 - exp(-\delta t/D_I) && \text{(S23)}\\
X_9^i(t) &\sim \text{binom}(X_8, p_{hosp}^i * (1 - p_{ICU}^i) && \text{(S24)}\\
X_{10}^i(t) &\sim \text{binom}(H^i(t), 1 - exp(-\delta t/D_H^i) && \text{(S25)}\\
X_{11}^i(t) &\sim \text{binom}(X_8, p_{hosp}^i * p_{ICU}^i) && \text{(S26)}\\
X_{12}^i(t) &\sim \text{binom}(H^i(t), 1 - exp(-\delta t/D_{ICU_1}^i) && \text{(S27)}\\
X_{13}^i(t) &\sim \text{binom}(H^i(t), 1 - exp(-\delta t/D_{ICU_2}^i) && \text{(S28)}\\
X_{14}^i(t) &\sim \text{binom}(H^i(t), 1 - exp(-\delta t/D_{ICU_3}^i) && \text{(S29)}\\
X_{15}^i(t) &\sim \text{binom}(X_7^i, 1 - p_{deathnonhosp}) && \text{(S30)}\\
X_{16}^i(t) &\sim \text{binom}(X_8^i, 1 - p_{deathnonhosp}) && \text{(S31)}\\
X_{17}^i(t) &\sim \text{binom}(X_{14}^i, 1 - p_{hosp}) && \text{(S32)}\\
X_{18}^i(t) &\sim \text{binom}(X_{14}^i, 1 - p_{deathicu}) && \text{(S33)}\\
X_{19}^i(t) &= X_7^i(t) - X_{15}^i(t) && \text{(S34)}\\
X_{20}^i(t) &= X_8^i(t) - X_{16}^i(t) && \text{(S35)}\\
X_{21}^i(t) &= X_9^i(t) - X_{17}^i(t) && \text{(S36)}\\
X_{22}^i(t) &= X_{14}^i(t) - X_{18}^i(t) && \text{(S37)}
\end{aligned}
$$

Table D. Compartments in the meta-population model.

Variable	Description
S	Susceptible
Ea	Exposed, will become asymptomatic
Es	Exposed, will become symptomatic
P	Presymptomatic infectious
A	Asymptomatic infectious
I	Symptomatic infectious
H	Hospitalized
ICU_1	In hospital will go to ICU
ICU_2	In ICU
ICU_3	Post-ICU care
R	Recovered and immune
D	Dead

S1.2.1 Model parameters

The main model parameters are described in Tables E and F. The probabilities of hospitalization, ICU admission and death are further adjusted by risk group and vaccination. The probabilities of hospitalization for the model can be found in Table H. Risk groups are discussed in Section SS2.3.

Table E. Parameters in the meta-population model.

Variable	Description	Value	Source
$\beta^i(t)$	Overall transmissibility	Fitted	
inf_s	Symptomatic infectiousness	1	Baseline
inf_a	Asymptomatic infectiousness	0.1	Feretti et al. [31]
inf_p	Presymptomatic infectiousness	1.25	Modified Feretti et al. [31]
D_E	Latent period	3 days	
D_P	Presymptomatic period	2 days	
D_A	Duration asymptomatic	4.8 days	
D_I	Duration symptomatic	4.8 days	

Table F. Age varying parameters in the meta-population model.

Variable	Description	0-9	10-19	20-29	30-39	40-49	50-59	60-69	70-79	80+
sus_i	Symptomatic Susceptibility	Fitted								
sus_a	Asymptomatic susceptibility	Fitted								
f_a	Fraction asymptomatic	0.4	0.4	0.4	0.4	0.4	0.4	0.4	0.4	0.4
f_s	Fraction symptomatic	0.6	0.6	0.6	0.6	0.6	0.6	0.6	0.6	0.6
D_H	Length of stay in Hospital	1.8	3.5	3.6	3.4	4.7	5.5	6.4	6.2	6.4
D_{ICU_1}	Length of stay in Hospital pre-ICU	1.4	1.4	1.4	2.8	3.7	3.1	3.9	4.5	4.5
D_{ICU_2}	Length of stay on ICU	3.3	3.3	3.3	3.3	9.3	9.5	16	15	14
D_{ICU_3}	Length of stay in hospital post-ICU	5	5	5	8.7	8.6	8.3	11	11	11
p_{icu}	Risk of ICU given hospitalization	Fitted								
$p_{deathnonhosp}$	Base Probability of death	Fitted								

S1.2.2 Regional contact matrix

The regional contact matrix is based on mobility data from Telenor [12] aggregated to the number of movements between the specified regions. We define the contact matrix such that amount of infectiousness that "leaks" out of a region is equal to the fraction of the population who traveled out of the region in one day. If the number of travelers from region i to region j is T_{ij} and the population in region i is P_i, then the regional contact matrix is given by:

$$M_{ij} = \frac{(P_i - \sum_j T_{ij})\delta_{ij} + T_{ij}(1 - \delta_{ij})}{P_i}, \tag{S38}$$

where δ_{ij} is the Kronecker delta symbol such that $\delta_{ij} = 1$ for $i = j$ and zero otherwise.

S1.2.3 Vaccination

As in the IBM, the vaccine effects for the different doses are given in Table B. In the MPM, we implement the time from receiving the first dose to getting the full effect of this dose as half the ramp-up time used in the IBM which corresponds to 14 days. Then the effect of the second dose is taken into account after the time interval between the doses of 84 days. The vaccination is implemented separately from the equations above as a constant moving of people from one meta-population i to another j at the appropriate time as determined by the vaccination strategy.

We also assume that the doses are equally distributed among everyone who has not yet been vaccinated including the people in other compartments than S, who will not receive any benefits.

S1.2.4 Seasonality and importation

Seasonality is implemented in the same way in the MPM model as in the IBM. Importation is implemented as shown in the model specification above by flipping on member of the S compartment to the I compartment. The amount of importation is identical to the IBM.

S1.2.5 Calibration

The calibration of the meta-population model follows the same overall approach as the IBM. We aim to simultaneously calibrate β-values and susceptibility values to the hospital incidence over time and to the age distribution of cumulative hospitalizations, shown in Fig G. The two targets are calibrated at the same time by assuming that the observed cumulative age distribution is constant over time and we define a log-likelihood for a given day t:

$$LL_t = \sum_i pois(X_t^i, prop^i O_t), \tag{S39}$$

where $pois$ is the Poisson distribution, X_t^i the modeled hospital incidence in age-group i on day t, $prop^i$ is the proportion of the cumulative hospitalization that is in age group i and O_t is the observed hospital incidence on day t.

From this log-likelihood we use a particle filter to better estimate the likelihood for the stochastic model. As for the IBM, we then use a Latin Hypercube Sampling (LHS) strategy for finding the maximum log-likelihood. We fit the 12 parameters by running 15,000 parameter values and estimating the log-likelihood by the particle filter with 15 particles. We choose the 10 parameter values with the highest log-likelihood. Fig H shows the distributions of estimated parameters.

We calibrate β and susceptibility for the actual strategy. For the other scenarios with a different configuration of municipalities into the regions in the model, we need modify the β values somewhat to get exactly the same epidemic trajectory as in the actual strategy. For each of the geographic patterns, we therefore fit a second scale-factor that is used to scale β such that the overall number of hospitalizations is on average the same as in the actual strategy when we have a baseline vaccination strategy without geographic prioritization.

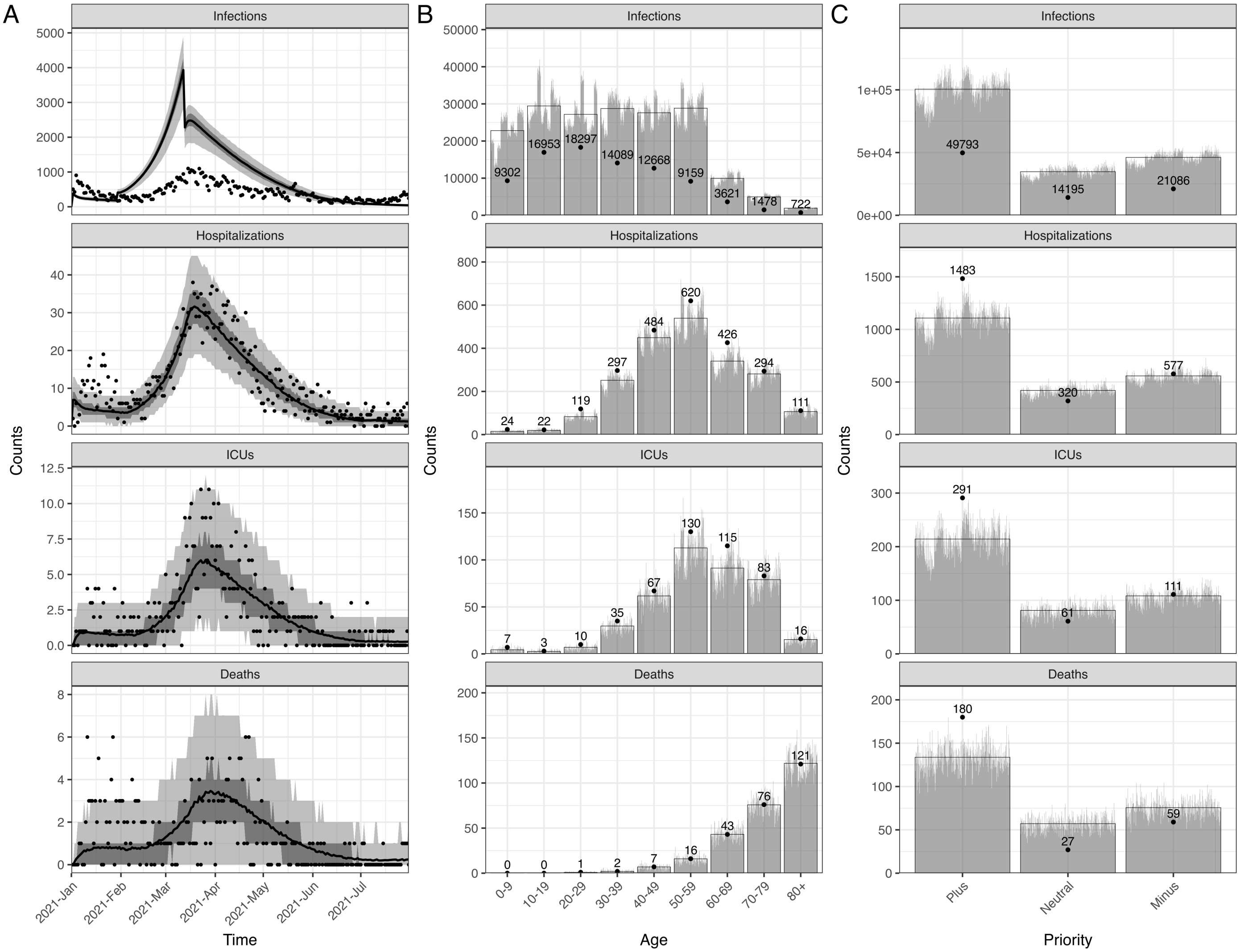

Fig G. The calibrated MPM and data. A: The time series data of all ages. The lines show the model fits with their 50 and 95% prediction intervals represented by gray areas. The dots show the observed data. B: The age distribution of total counts. C: The geographic-specific priority distribution of total counts. The gray bars show the counts of each simulation and the full bars with black borders show the mean of all simulations. The data are shown in dots with their exact numbers.

The age-specific probabilities of death and ICU admission are calculated following the same approach as the IBM. Table G show the age-specific probabilities of death and ICU admission.

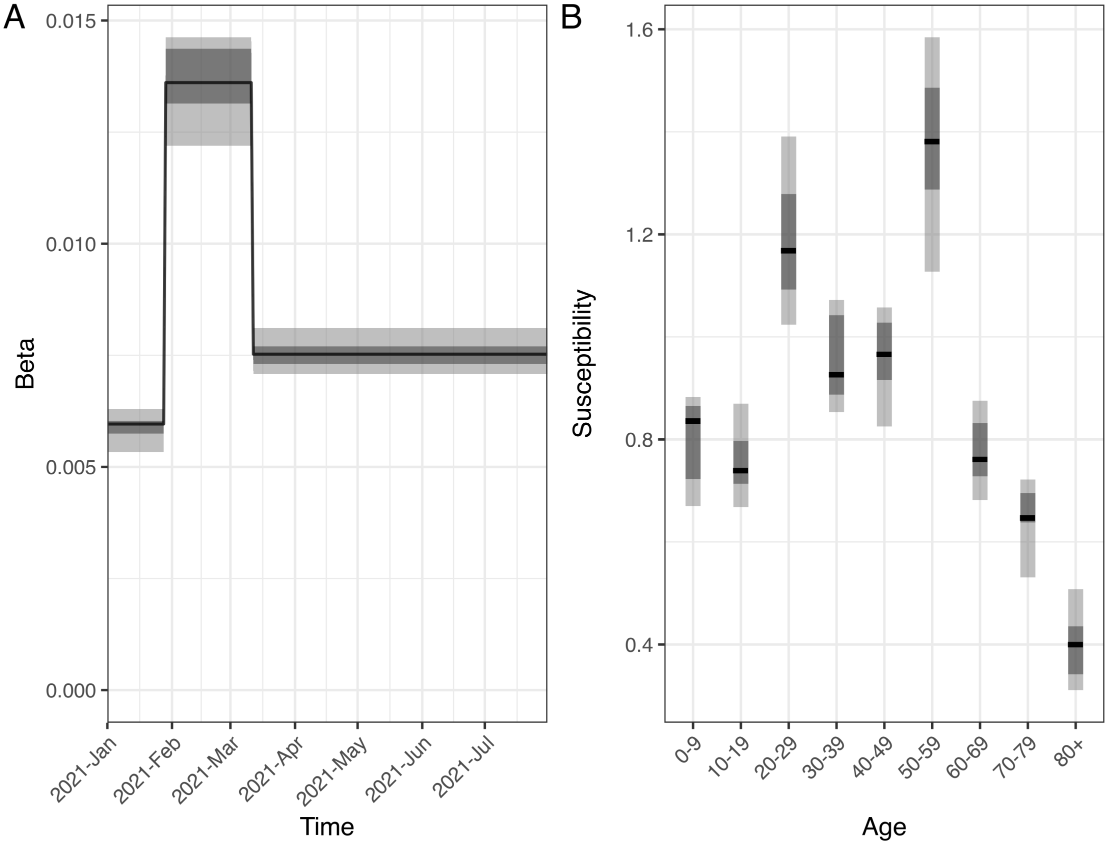

Fig H. The marginal distributions of 12 estimated parameters in the MPM. (A) Beta in 3 time periods and (B) susceptibilities of 9 age groups are sampled from Latin hypercube sampling (LHS) approach. The black lines show the median values, and the darker (lighter) gray areas show the ranges of 25th and 75th percentiles, i.e. lower and upper quantiles (2.5th and 97.5th percentiles) of the estimated parameters.

S2 Model assumptions

There are some assumptions shared between 2 models.

S2.1 Initial condition

The initial conditions for the two models (IBM and MPM) were taken from the situational awareness model run by the Norwegian Institute of Public Health (NIPH) [32–34]. In this model, we estimated the numbers in each of the epidemiological compartments for each county in Norway based on calibration to hospital incidence. To get initial conditions on the municipality level, we distributed the county level data by population.

Table G. The age-specific probabilities of death given infection and ICU admission given hospitalization in the MPM.

	Probabilities of death (%)		Probabilities of ICU admission (%)
**Age groups**	**Without risk factors**	**With risk factors**	**All population**
0-9	0.00006	0.0001	29.2
10-19	0.00001	0.00002	13.6
20-29	0.0008	0.001	8.4
30-39	0.001	0.002	11.8
40-49	0.007	0.01	13.8
50-59	0.03	0.05	21.0
60-69	0.2	0.4	27.0
70-79	1.5	2.7	28.2
80+	9.4	17.3	14.4

We assume that the probabilities of death depend on both age and risk status while the probabilities of ICU admission depend on age only. The age-specific probabilities of death given infection are calculated based on the observed number of deaths and the estimated mean number of infections. The age-specific probabilities of ICU admission given hospitalization are calculated based on the observed numbers of ICU admissions and hospitalizations.

S2.2 Relative regional reproduction numbers

It is very difficult to accurately estimate the reproduction number in each municipality due to small number of cases. We therefore estimate and approximate relative reproduction numbers per municipality based on the total number of confirmed cases between February 2020 and May 2021. The aim of the method is to determine what factor we need to scale the estimated national reproduction numbers to get the observed fraction of infected people in each municipality as compared to the national fraction.

Since there are many small municipalities in Norway where a small outbreak can significantly affect the overall fraction of infected, we first do a simple partial-pooling analysis where we fit a simple Bayesian model of the number of observed cases in each municipality, k_i by estimating a fraction, f_i and combining it with the population in the municipality, p_i. We use an hierarchical prior on the fractions, f_i to achieve the partial pooling effect.

$$k_i \sim \mathrm{NB}(p_i * f_i, \frac{1/1000}{p_i}) \quad (S40)$$

$$f_i \sim \mathrm{Beta}(\alpha, \beta) \quad (S41)$$

$$\alpha \sim \mathcal{N}(1, 0.4) \quad (S42)$$

$$\beta \sim \mathcal{N}(100, 20) \quad (S43)$$

The model was implemented in the Stan language [35] using the rStan interface [36].

Once we have an estimated partially-pooled fraction f_i for each municipality we implemented a simple SEIR model with change points for the beta-parameter with values corresponding to the estimated reproduction number in the NIPH situational awareness model [32, 33]. The SEIR model was a simplified version of the national model described here [34]. The model had symptomatic, presymptomatic and asymptomatic transmission and was seeded by data on imported cases in Norway. We assumed a detection probability of 55%.

For each of the fractions, f_i, we found the scale factor s_i which if applied to all the beta-values in the national model would have given a national fraction of infections equal to f_i. The reproduction numbers are then normalized to be equal to or less than 1, shown in Fig IC.

In the IBM, these 356 relative reproduction numbers are used directly, while in the MPM they have to be aggregated since the model uses fewer regions. We use a population-weighted average of the individual scale factor to estimate the relative reproduction number for these combined regions. As mentioned in the MPM section, this aggregation does not perfectly preserve the overall reproduction number, but this is fixed in the calibration step.

S2.3 Probabilities of hospitalization

We used the meta-analysis [37] for probabilities of needing hospitalization in each age group as the base values. We increased the probability for those 0-9 years old to the level for those 10-19 years old due to higher observed hospital admissions in Norway. We reduced the probability for those over 80 years old by 25% since this proportion of elderly lived in assisted care homes or nursing homes, and they were generally not admitted to hospital for COVID-19 but treated in the facilities in Norway.

As the main wave during the simulation period (January - July 2021) was contributed by the Alpha variant, we used the age-specific adjusted risk ratio of the Alpha variant compared to wild-type [25] on top of the above probabilities of hospitalization. We noted that the risk ratio could be overestimated compared to other studies in the UK [38, 39], Denmark [40] and some European countries [41], and we thus applied a factor of 0.5 for the probability of hospitalization. In the absence of seroprevalence data, it was difficult to identify the true infection-hospitalization ratio as other studies for example in the Netherlands and Portugal [21, 22]. Here, we included both scenarios of applying the 0.5 factor and without it. The latter was considered as a sensitivity analysis. The corresponding case detection fractions (i.e. the ratios of observed cases from the data and infections from the model) could be low and high, supported by two independent studies (one in January 2021 [42–44] and one in November/December 2020 [45]), respectively.

Furthermore, based on an analysis of Norwegian data from emergency registry Beredt C19 [46], we found that people in risk groups had a 2.7 times higher likelihood of hospitalization and a 1.8 higher likelihood of death [47]. The analysis was based on data until November 2020 and compared the probability of hospitalization and death among those who tested positive based by risk group adjusting for age. Based on the relative sizes of the risk groups, we estimated the risk of hospitalization. Table H shows the age-specific probabilities of hospitalization and all the scale factors.

Table H. The probabilities of hospitalization.

Age (years)	Meta-analysis (%)	Norway	RR	Halved	Total (%)	Risk (%)	Non-risk (%)
0-9	0.1	2	1.2	0.5	0.12	0.29	0.11
10-19	0.2	1	1.2	0.5	0.12	0.29	0.11
20-29	0.5	1	2.1	0.5	0.525	1.28	0.47
30-39	1	1	3.1	0.5	1.55	3.70	1.37
40-49	2	1	3.1	0.5	3.1	6.95	2.57
50-59	4	1	2.0	0.5	4	8.06	2.99
60-69	9	1	1.8	0.5	8.1	14.16	5.25
70-79	20	1	1.6	0.5	16	23.79	8.81
80+	30	0.75	1.6	0.5	18	23.86	8.84

The age-specific probabilities of hospitalization were taken from the meta-analysis [37], of which the values of youngest and oldest age groups were modified to match the situation in Norway. The total probabilities were scaled by the halved risk ratio (RR) of the Alpha variant compared to the wild-type [25]. The probabilities for the risk groups were 2.7 times higher [47], and those for non-risk groups were adjusted to the lower values accordingly.

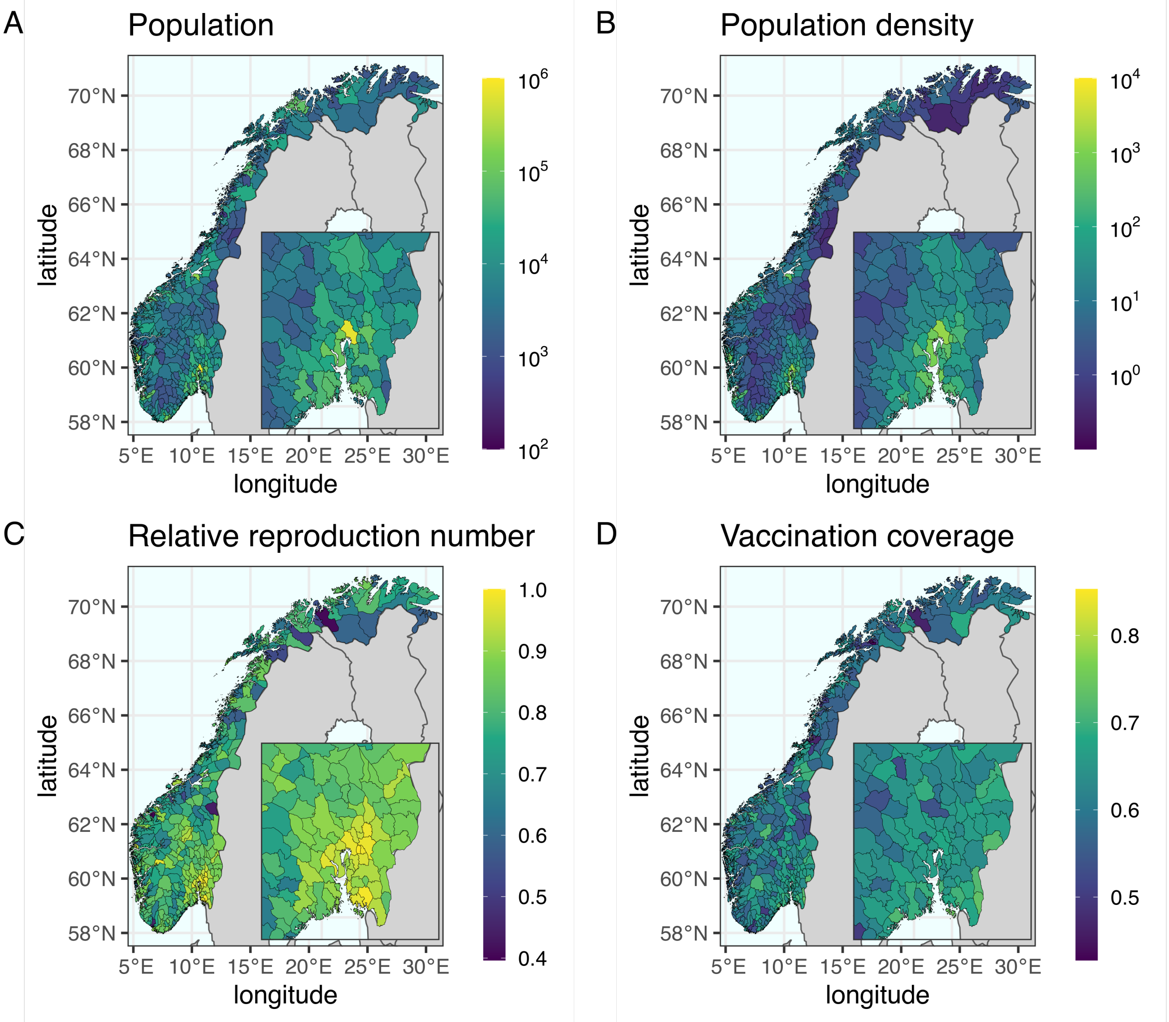

Fig I. The population, density, relative reproduction number and vaccination coverage of 356 municipalities in Norway. A: The population size in log10-scale illustrates a highly heterogeneous geographic distribution. The population size in Oslo is about 700,000. B: The population density in log10-scale. The population density in Oslo is about 1500 per km2. C: The relative reproduction number is normalized with a maximum at 1. Oslo and its surrounding municipalities are the ones with the highest numbers. D: The vaccination coverage of the 1st dose. The overall coverage in Norway at the end of July 2021 was about 64%. The maps were created using two R packages: "rnaturalearth" for country-level data and "fhidata" for Norwegian municipality-level data. All open-source shape files are licensed under Creative Commons BY 4.0 (CC BY 4.0) and CC0 1.0 (No Copyright), respectively. The country-level data is sourced from Natural Earth (`https://www.naturalearthdata.com`), while the Norwegian municipality-level data was obtained from George (`https://kartkatalog.geonorge.no/metadata/norske-fylker-og-kommuner-illustrasjonsdata-2020-(klippet-etter-kyst)/7408853f-eb7d-48dd-bb6c-80c7e80f7392`).

S3 Data

There are mainly three sets of Norwegian data used in this study taken from Emergency preparedness register for COVID-19 (Beredt C19) [46].

S3.1 Registry data

There were in total 86289 confirmed cases, 2397 hospital admissions, 466 ICU admissions and 266 deaths registered and collected by Norwegian Surveillance System for Communicable Diseases (MSIS) [48] and the Norwegian Intensive Care and Pandemic Registry (NIPaR) [49, 50] from January to July 2021. Fig J shows the daily numbers with rolling averages, cumulative numbers, geographic distribution and age distributions of the 4 outcomes. Cases are defined as people who tested positive with a PCR test in Norway by date of testing. Hospitalizations include all admissions where COVID-19 was the main cause for the admission as determined by the doctor and where the date of admission and testing positive was after the 1st of January 2021. The number of ICU admissions are patients who tested positive and where admitted to an ICU after the 1st of January 2021. Deaths are COVID-19 associated deaths, defined as deaths reported by doctors as due to COVID-19 or as deaths with COVID-19 as the cause of death on the death certificate. We included COVID-19 associated deaths where the date of test and date of death was after the 1st of January 2021.

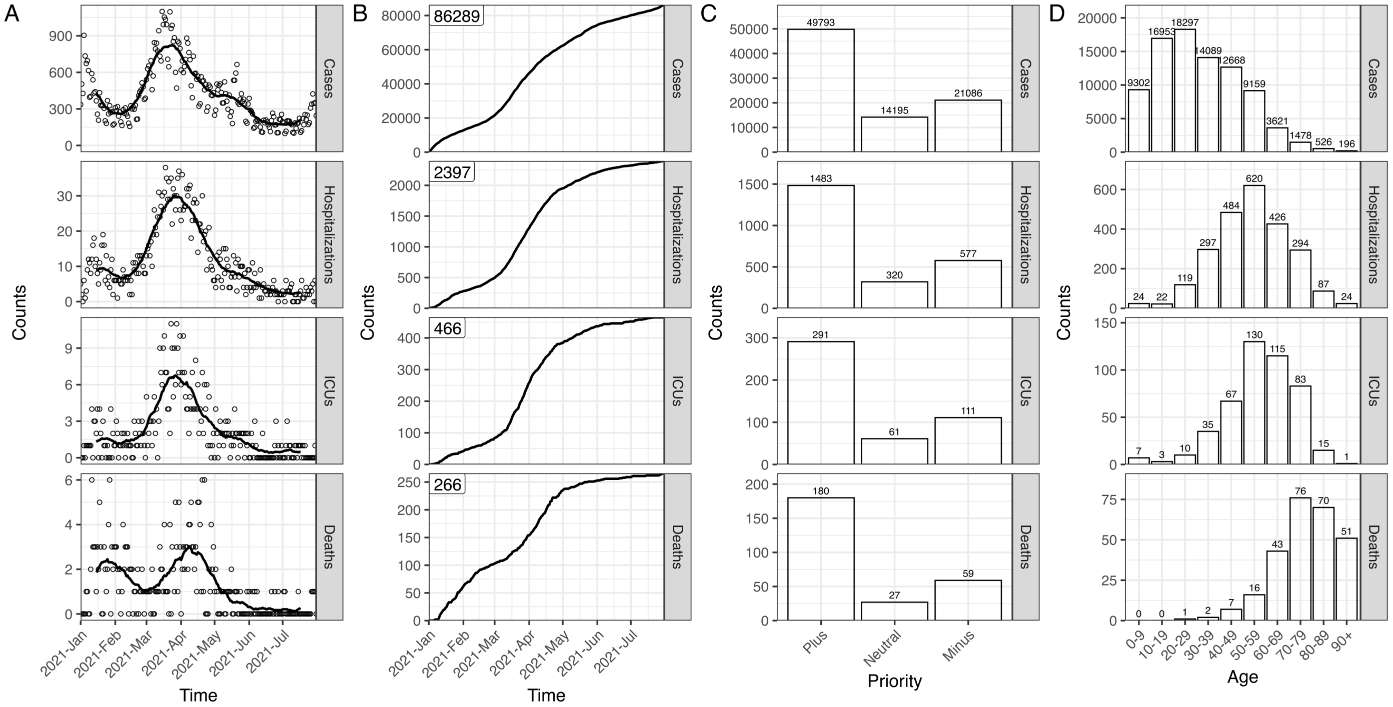

Fig J. The observed data from January to July 2021 in Norway. A: The daily numbers (circles) and rolling averages (lines). B: The cumulative numbers. C: The priority distributions of geographic regions. D: The age distributions of confirmed cases, hospitalizations, ICU admissions and deaths are shown in each panel.

S3.2 Test data

The test positive data were reported by medical doctors and collected by Norwegian Surveillance System for Communicable Diseases (MSIS) [48]. The data from January to July 2021 shows that 45.1%, 38.6%, 9.9% and 6.2% of the cases were infected within households, in community, workplaces and schools, respectively. Community transmission refers to all routes (e.g. public event, private arrangement, prison, health institution, organized leisure activity, private event in public place, travel, collection in private homes, restaurant, bar, nightclub and public transport) other than households, schools or workplaces as the categorization in the individual-based model (IBM). Additionally, we note that the data of importation used in both models are shown Fig BF.

S3.3 Vaccination data

SYSVAK is a national, electronic immunization registry that records an individual's vaccination status and vaccination coverage in Norway [51]. The data were aggregated by date, municipality, age and risk status. Fig KA shows the total number of first doses distributed from January to July in 2021 in Norway is about 3.5M. Fig KB shows the age-specific coverage from January to July in 2021, and Fig ID shows the geographic distribution of coverage. The models used the normalized data according to the synthetic population to match with the vaccination coverage.

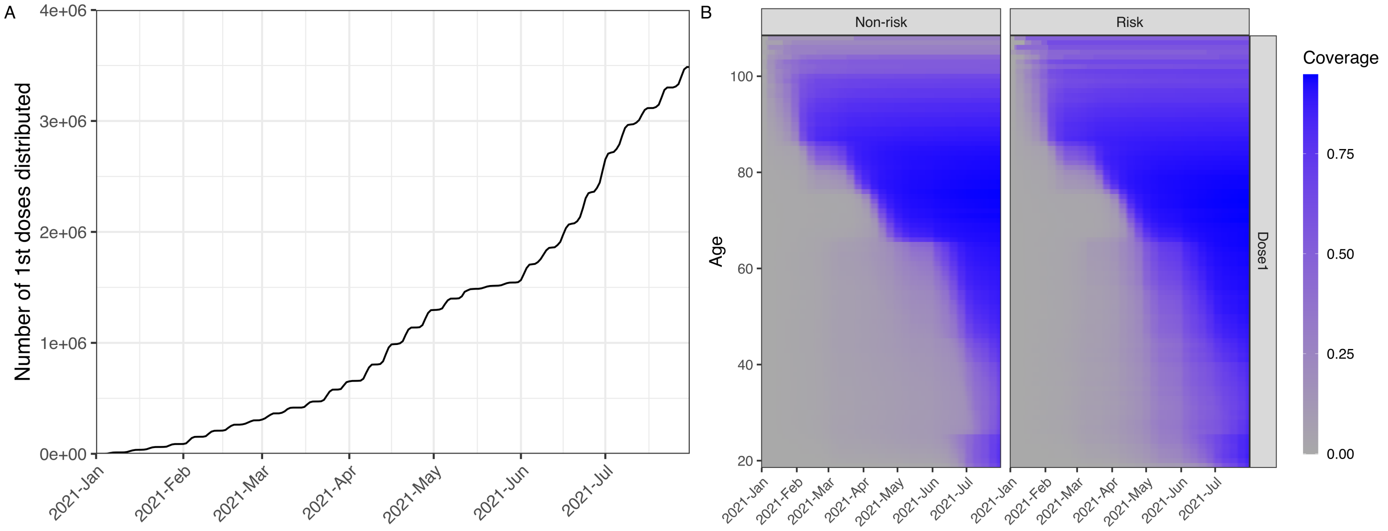

Fig K. The vaccination data from SYSVAK. A: The cumulative number of first doses distributed nationally. The number of first doses per day taken from historical data is shared in all scenarios. B: The age- and risk-specific vaccination coverage. The coverage of each group from low to high is shown by color from gray to blue. The age prioritization is shown in Table J.

S4 Vaccination strategies

The distribution of all vaccine doses follows a prioritization process. Firstly, doses are allocated to different regions based on geographic prioritization, and secondly, within each region, doses are prioritized based on age (and risk) of individuals.

Fig L illustrates the distribution of relative reproduction numbers for municipalities grouped by priority level (*Plus*, *Minus*, and *Neutral*) for each of the 15 selection strategies. The original selection strategy ($\Delta n = 0$) is based on the real

implemented step-2 geographic prioritization plan in Norway, from the 19th of May 2021 [52]. The *Plus* group comprises municipalities with the highest regional reproduction numbers, while the *Neutral* group may not necessarily be located between the *Plus* and *Minus* groups. Table I presents the number of municipalities and their corresponding adult population fractions in each priority group.

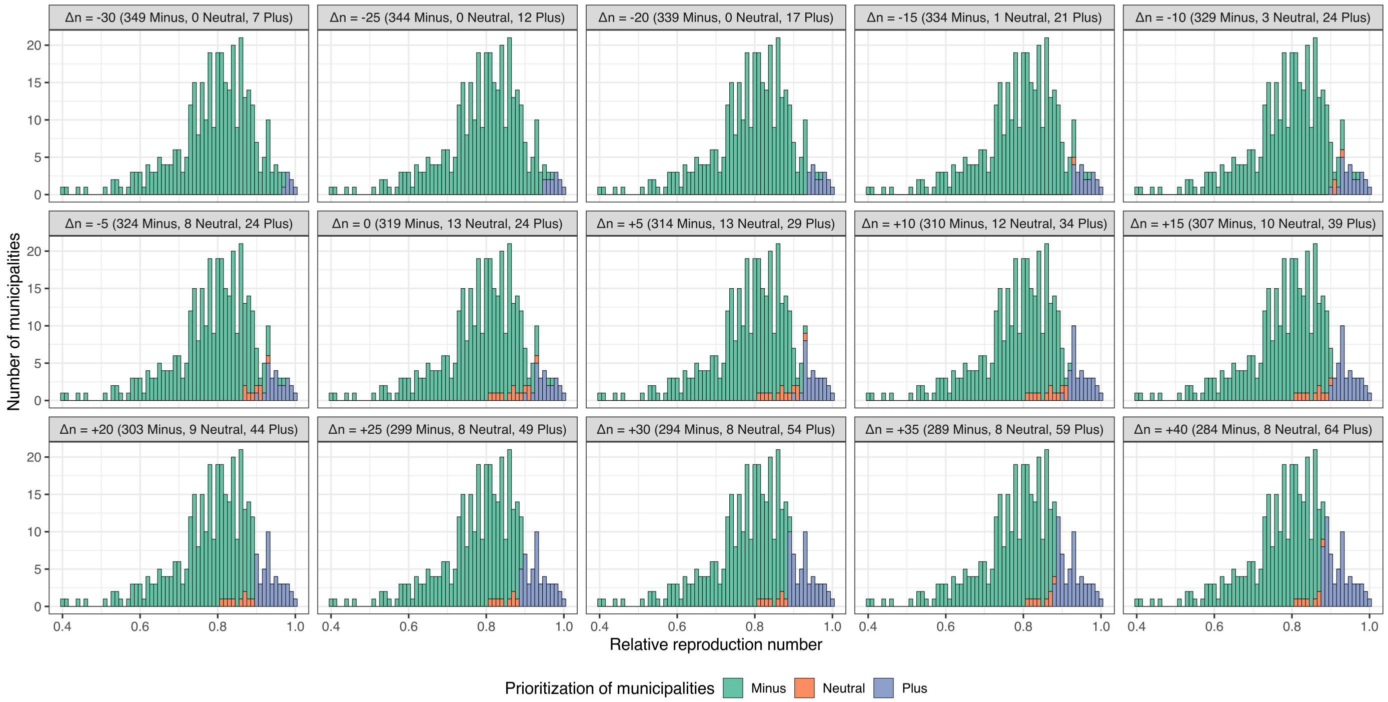

Fig L. The relative reproduction number distribution of municipalities in alternative strategies. 356 municipalities with their own relative reproduction numbers are classified into 3 groups: *Minus*, *Neutral* and *Plus* colored in green, orange and blue. Each strategy has different numbers of municipalities in the three groups. The original prioritization ($\Delta n = 0$) is the selection in the real implementation. The index of moves of municipality priority Δn is shown in brackets, and the number of municipalities in each group are shown accordingly on each panel title.

Fig M shows the total number of prioritized vaccine doses shifted from the *Minus* to the *Plus* group for all alternative strategies. The most extreme strategy ($\Delta p = 300\%$ and $\Delta n = -25$) prioritizes 3 million doses to 12 municipalities in the *Plus* group. However, for each Δn, there exists a maximum threshold of Δp, as shown in Table I. The smaller population size in the *Minus* group limits the total number of doses that can be prioritized to the *Plus* group. The percentage of extra prioritized doses can only reach a maximum of $\Delta p = 70\%$ by moving 40 municipalities from the *Minus* group ($\Delta n = +40$) in one extreme direction. Conversely, prioritized doses can reach up to $\Delta p = 300\%$ by moving 30 municipalities from the *Plus* group ($\Delta n = -30$) in another extreme direction.

Table J shows the age prioritization categories for different risk groups and their corresponding population sizes. Within each region, vaccines are distributed according to these priority groups. However, in reality, a portion of vaccine doses are allocated to healthcare workers, who face higher risk due to their front-line roles, while simultaneously prioritizing the elderly. The NIPH modeling report [53] determined the age prioritization for the general population. Notably, those aged 25-39 years old are the lowest priority group, after individuals aged 18-24 years old.

Fig N illustrates the age-based trade-offs resulting from different geographic prioritization strategies. The percentage

Table I. The characteristics of the shifts of municipality priority Δn.

	**Number of municipalities**			**Adult population fraction (%)**			**Population ratio (%)**
Δn	*Minus*	*Neutral*	*Plus*	*Minus*	*Neutral*	*Plus*	*Minus / Plus*
-30	349	0	7	80.5	0.0	19.5	412
-25	344	0	12	76.3	0.0	23.7	321
-20	339	0	17	72.7	0.0	27.3	265
-15	334	1	21	69.5	0.4	30.1	230
-10	329	3	24	61.6	6.8	31.6	194
-5	324	8	24	56.9	11.5	31.6	180
**0**	**319**	**13**	**24**	**48.0**	**20.4**	**31.6**	**151**
+5	314	13	29	47.7	20.4	31.9	149
+10	310	12	34	46.6	19.9	33.4	139
+15	307	10	39	44.8	13.5	41.7	107
+20	303	9	44	44.3	13.0	42.7	103
+25	299	8	49	43.6	12.5	43.9	99
+30	294	8	54	42.9	12.5	44.6	96
+35	289	8	59	42.5	12.5	45.0	94
+40	284	8	64	41.0	12.5	46.5	88

For each Δn, the table shows the number of municipalities, their corresponding adult population fraction in *Minus*, *Neutral* and *Plus* groups and the population ratio of *Minus* and *Plus*.

Table J. The age prioritization in different risk categories.

**Priority order of risk categories**	**Population**
All aged 85+	116995
All aged 75-84	290097
All aged 65-74	534724
All healthcare workers	322994
People with underlying diseases, aged 55-64	164691
People with underlying diseases, aged 45-54	119524
People with underlying diseases, aged 18-44	154318
Other people, aged 55-64	422891
Other people, aged 45-54	563932
Other people, aged 18-24 and 40-44	662403
Other people, aged 25-39	896401

The priority of each risk category is placed in order. The oldest age group is given the highest priority among all while those aged 25-39 years without underlying diseases are the least. Their approximate population are shown accordingly.

of extra doses (Δp) is varied while municipality priority moves are fixed ($\Delta n = -25$). The geographic prioritization of vaccine distribution can ultimately influence age-based prioritization. For instance, without geographic prioritization ($\Delta p = 0$), all individuals aged 80 years and above can receive their first dose by March. Conversely, under the most extreme strategy ($\Delta p = 300\%$), they can receive it by April. There are switches between strategies for other age groups, such as in mid-February and early April for those aged 70-79 and 60-69 years old, respectively. It is important to note

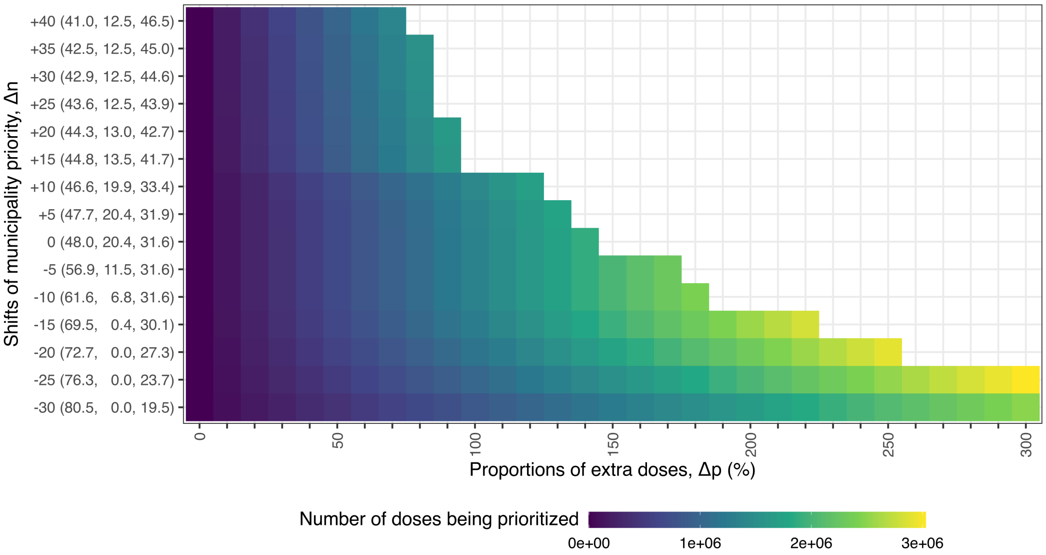

Fig M. The number of prioritized doses distributed from the *Minus* group to the *Plus* group in the alternative strategies. The color shows the number of doses from low to high by color blue to yellow. The baseline strategy without geographic prioritization on the first column ($\Delta n = 0\%$) refers to no doses being prioritized. The y-axis shows the population fractions (%) of three groups (*Minus*, *Neutral*, and *Plus*) for each shift in municipality priority (Δn). The geographic distribution of municipality priority (Δn) can be found in Fig 2. The maximum thresholds of Δp can be found in Table I.

that the results from the IBM refer to the timing of vaccination, while those from the MPM indicate the timing of vaccine effectiveness, resulting in a time delay between the two models.

S5 The scenario without vaccination

We considered a scenario without vaccination to show the effect of the mass vaccination program. In the absence of vaccination, as shown in Table K, all infections, hospitalizations, ICU admissions, and deaths were higher than in scenarios where vaccination was implemented. All RRR were negative. In the IBM, the mean RRR (and their 95% CIs) were -5.8 (-6.3 to -5.4)%, -17.2 (-17.7 to -16.7)%, -20.0 (-20.7 to -19.4)%, and -80.7 (-82.1 to -79.3)%, corresponding to 13,734 (12,723 to 14,746) more infections, 455 (443 to 469) more hospitalizations, 103 (100 to 106) more ICU admissions, and 190 (187 to 194) more deaths than the baseline strategy, respectively. In the MPM, the mean RRR (and their 95% CIs) were -13.5 (-13.9 to -13.1)%, -26.4 (-26.9 to -25.9)%, -28.7 (-29.4 to -27.9)%, and -106.0 (-107.0 to -104.0)%, corresponding to 26,246 (25,476 to 27,017) more infections, 574 (563 to 586) more hospitalizations, 118 (115 to 121) more ICU admissions, and 252 (249 to 255) more deaths than the baseline strategy, respectively. The effective reproduction number from the IBM is shown in Fig S.

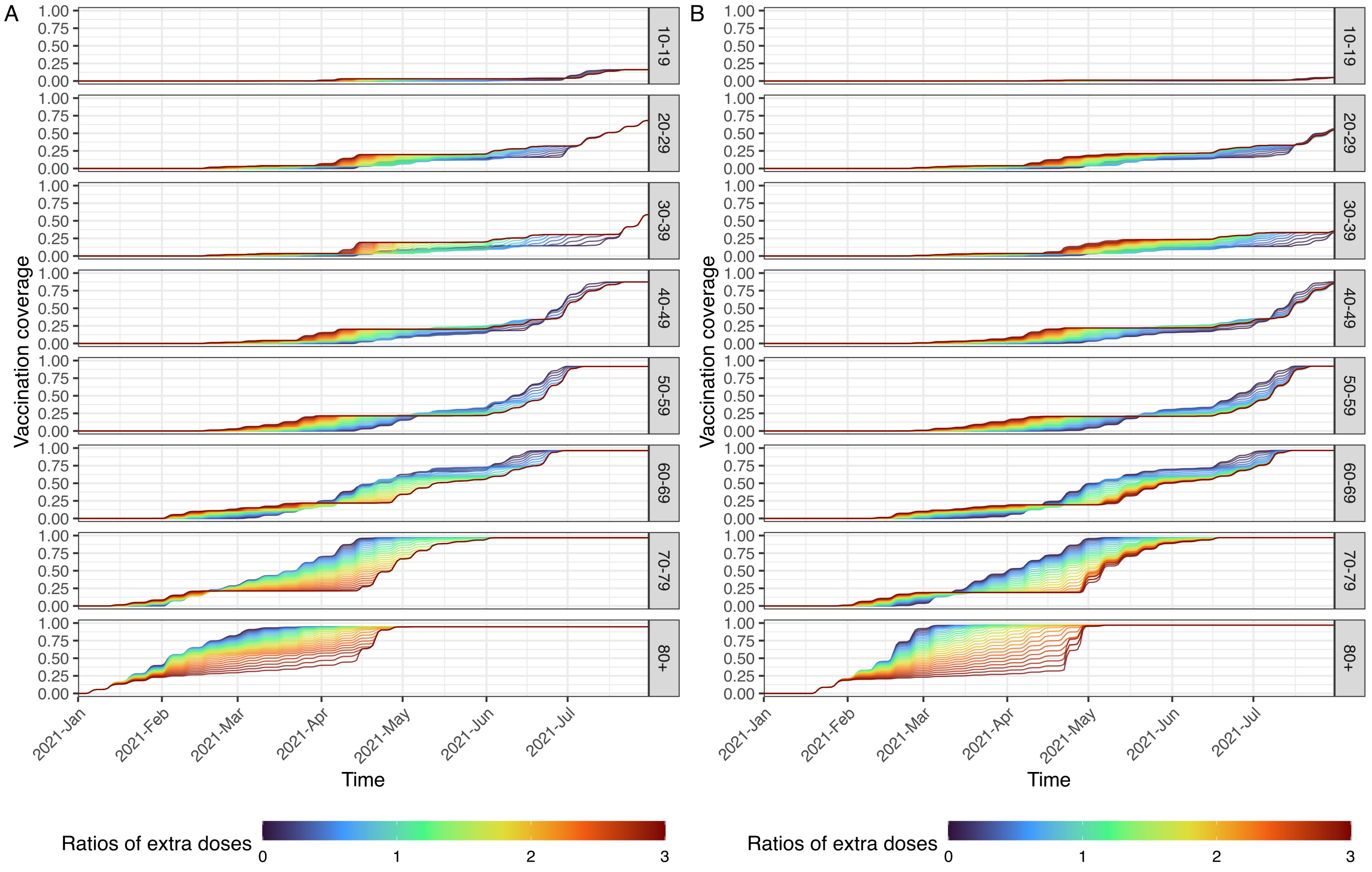

Fig N. The age-specific vaccination coverage in the alternative strategies. Each line in different colors shows a strategy of alternative ratio of extra doses (Δp) given fixed moves of municipality priority ($\Delta n = -25\%$). The blue and red color present lower and higher levels of geographic prioritization, respectively. Given a higher level of geographic prioritization, the priority of the older people is lower, for example fewer doses are distributed to those above 80 years old during the first few months. A: The timing of vaccination in the IBM. B: The timing of vaccine protection activated in the MPM. Note that there is a 2-week time delay from vaccination to providing protection in the MPM.

S6 Additional results in the alternative strategies

Fig O and Fig P show the geographic- and age-specific RRR from both IBM and MPM in the alternative strategies, respectively. Fig Q and Fig R show the RRR from both IBM and MPM in the alternative starting time, respectively.

Fig S shows the effective reproduction numbers from the IBM. We note that the reproduction numbers are the case reproduction numbers instead of instantaneous reproduction numbers [54–56]. Four scenarios (i) the actual strategy, i.e. the reality; (ii) the scenario without vaccination; (iii) the baseline strategy without geographic prioritization; and (iv) the optimal strategy ($\Delta n = -25, \Delta p = 300\%$) for minimizing infections as shown in the main text are included.

Fig T and Fig U show the comparison of two models in the actual strategy and optimal strategy for minimizing infections, respectively. Fig U shows the comparison in the geographic difference on top of the trends and age

Table K. The relative risk reduction (RRR) of different strategies compared to the baseline strategy without geographic prioritization.

		No vaccination	Best strategy
**Models**	**Outcomes**	**RRR, % (95% CI)**	**RRR, % (95% CI)** [Δp, Δn]
IBM	Infections	-5.8 (-6.3, -5.4)	16.4 (16.1, 16.7) [300%, -25]
IBM	Hospitalizations	-17.2 (-17.7, -16.7)	19.4 (19.0, 19.7) [250%, -20]
IBM	ICUs	-20.0 (-20.7, -19.4)	19.2 (18.8, 19.6) [250%, -20]
IBM	Deaths	-80.7 (-82.1, -79.3)	8.0 (7.5, 8.6) [100%, $+10$]
MPM	Infections	-13.5 (-13.9, -13.1)	16.0 (15.7, 16.3) [300%, -25]
MPM	Hospitalizations	-26.4 (-26.9, -25.9)	15.4 (15.0, 15.7) [300%, -25]
MPM	ICUs	-28.7 (-29.4, -27.9)	15.6 (15.1, 16.1) [300%, -25]
MPM	Deaths	-106.0 (-107.0, -104.0)	5.5 (4.9, 6.2) [70%, -15]

The mean RRR (and their 95% confidence intervals) of the scenario without vaccination and the best strategies are shown in the two columns. The best strategies, selected separately for each health outcome, are shown in square brackets.

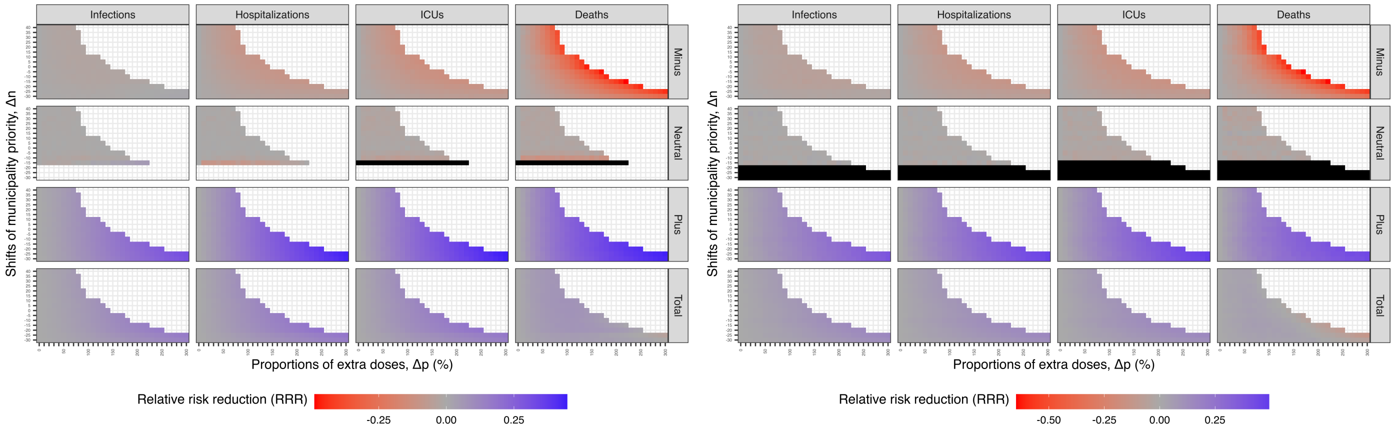

Fig O. The geographic-specific mean RRR from both (Left) IBM and (Right) MPM in the alternative strategies. The RRR is calculated for each 3 geographic-prioritization groups and 4 health outcomes on infections, hospitalizations, ICU admissions, and deaths in alternative selection of municipality priority and level of geographic prioritization. The black color represents unidentifiable RRR values due to low influence or the absence of municipalities in *Neutral* group. The total RRR on the bottom row shows the total of all geographic groups.

distributions in Fig 6 in the main text. In general, there are more counts, especially infections, hospitalizations and ICU admissions, in the *Plus* group in the IBM than MPM.

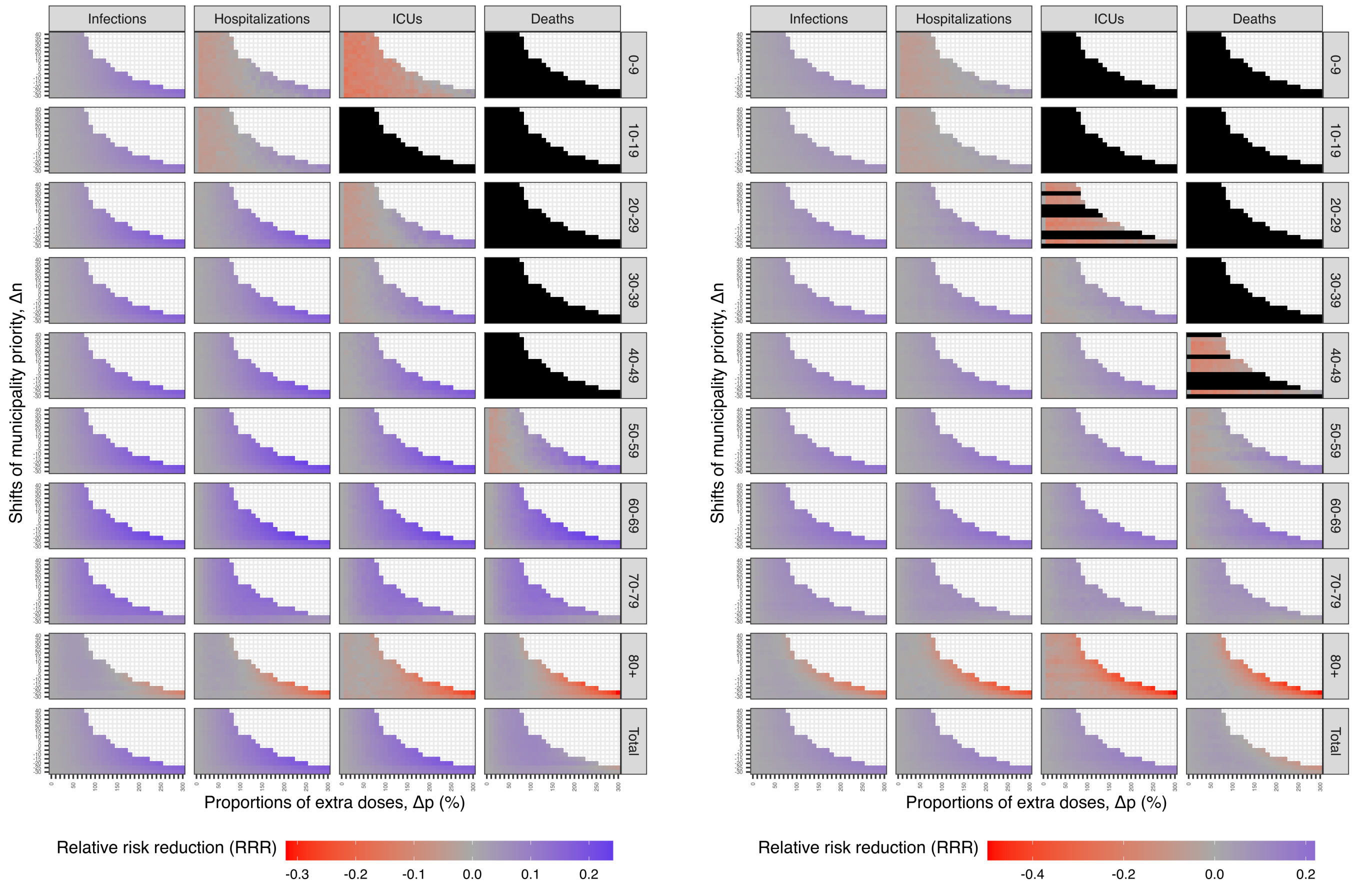

Fig P. The age-specific mean RRR from both (Left) IBM and (Right) MPM in the alternative strategies. The RRR is calculated for each 9 age groups and 4 health outcomes on infections, hospitalizations, ICU admissions, and deaths in alternative selection of municipality priority and level of geographic prioritization. The black color represents unidentifiable RRR values due to low influence of severe outcomes among the younger population. The total RRR on the bottom row shows the total of all ages.

S7 Sensitivity analysis

In this sensitivity analysis, the entire analysis was performed without the factor of 0.5 on the probabilities of hospitalization. We found that our results are not sensitive to the factor of 0.5 on the probabilities of hospitalization by obtaining similar results in both IBM and MPM.

Both models are re-calibrated that 12 free parameters were estimated by fitting to the daily and age-specific hospital admissions using Latin hypercube sampling (LHS) and least squares method. Tables L and M show the age-specific probabilities of death given infection from two models, respectively. The probabilities calculated based on the ratios of observed number of deaths to the estimated mean number of infections. Fig VA and Fig WA show the distributions of calibrated parameters and the fits to observed data from the IBM. Fig VB and Fig WB show the ones from the MPM.

The relative risk reduction (RRR) of alternative strategies were also calculated accordingly. Fig X shows the results

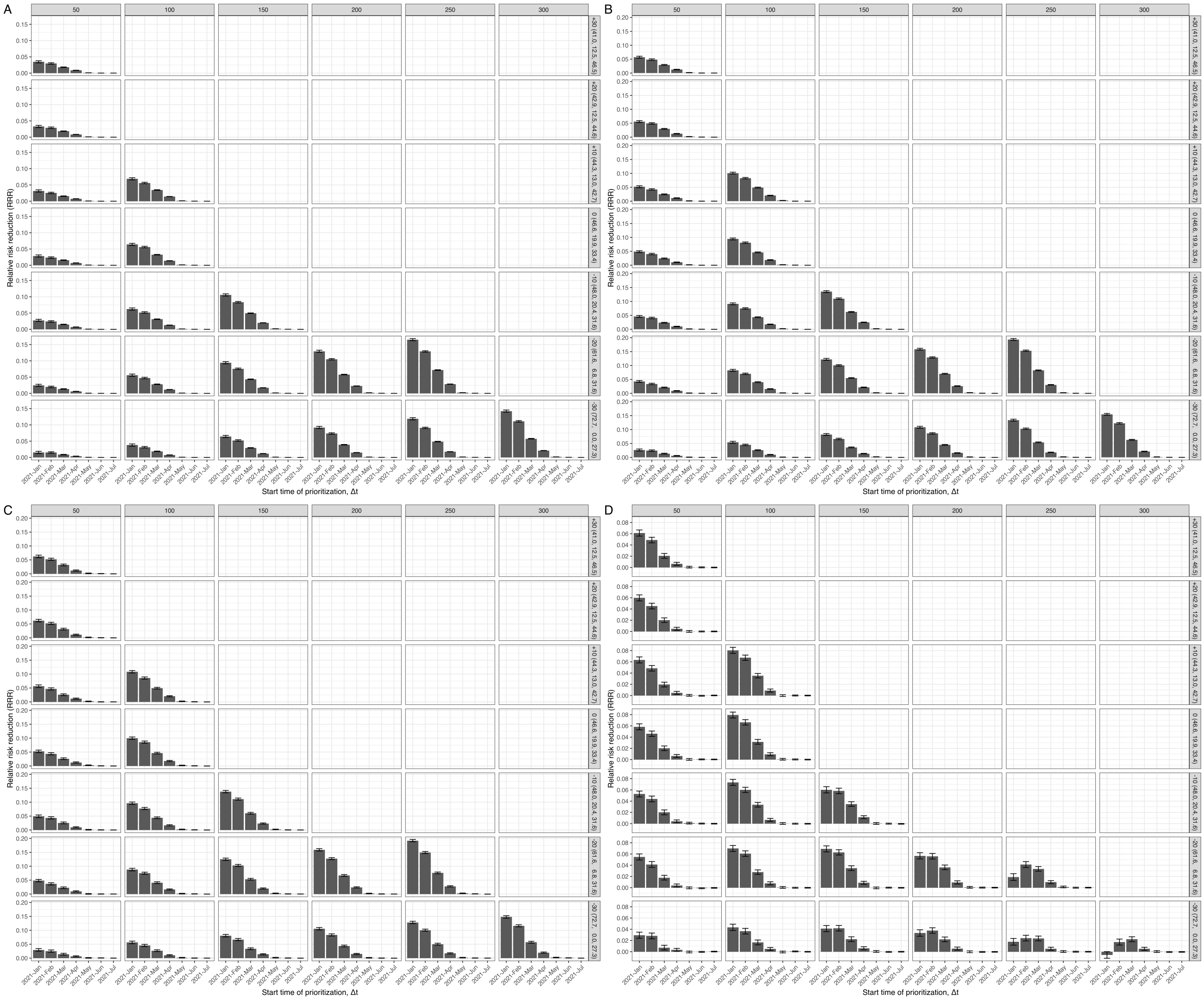

Fig Q. The RRR in the alternative starting time for the IBM. Each panel shows the RRR for (A) infections, (B) hospitalizations, (C) ICU admissions, and (D) deaths. While the RRR for infections, hospitalizations, and ICU admissions decrease by starting time across all strategies, the RRR of deaths decrease by starting time only when $\Delta p < 200\%$. The bars show the mean values and their 95% confidence intervals using $\pm 1.96 \frac{\sigma}{\sqrt{N}}$, where σ is standard deviation of RRR and $N = 1000$ is the number of simulations. The benefits are limited if the geographic prioritization started after May 2021.

from the IBM and MPM in alternative strategies of geographic prioritization. Table N shows all the RRR of the scenario without vaccination and the best strategies from two models. All the RRR are similar to the original analysis.

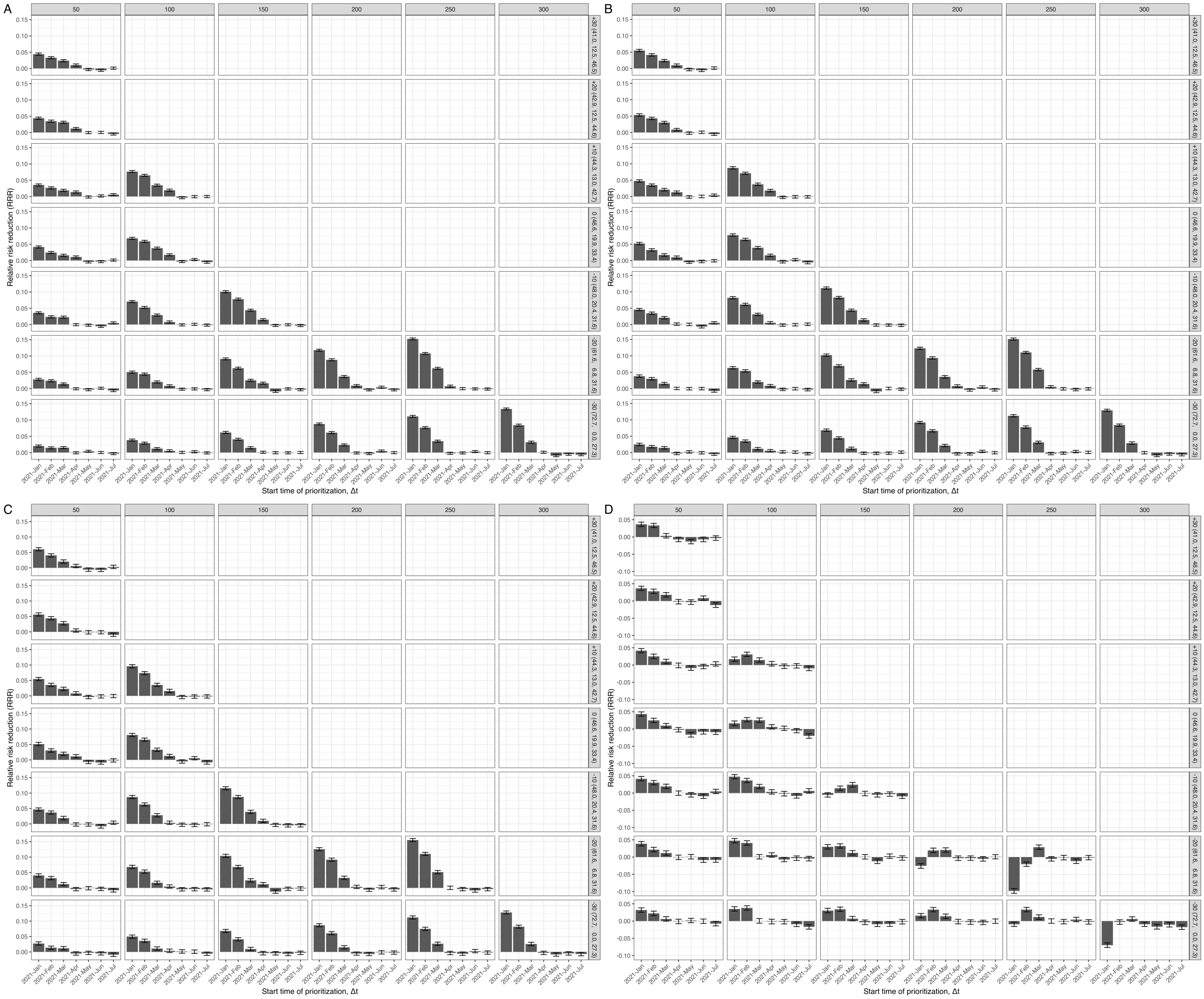

Fig R. The RRR in the alternative starting time for the MPM. Each panel shows the RRR for (A) infections, (B) hospitalizations, (C) ICU admissions, and (D) deaths. While the RRR for infections, hospitalizations, and ICU admissions decrease by starting time across all strategies, the RRR of deaths decrease by starting time only when $\Delta p < 200\%$. The bars show the mean values and their 95% confidence intervals using $\pm 1.96 \frac{\sigma}{\sqrt{N}}$, where σ is standard deviation of RRR and $N = 1000$ is the number of simulations. The benefits are limited if the geographic prioritization started after May 2021.

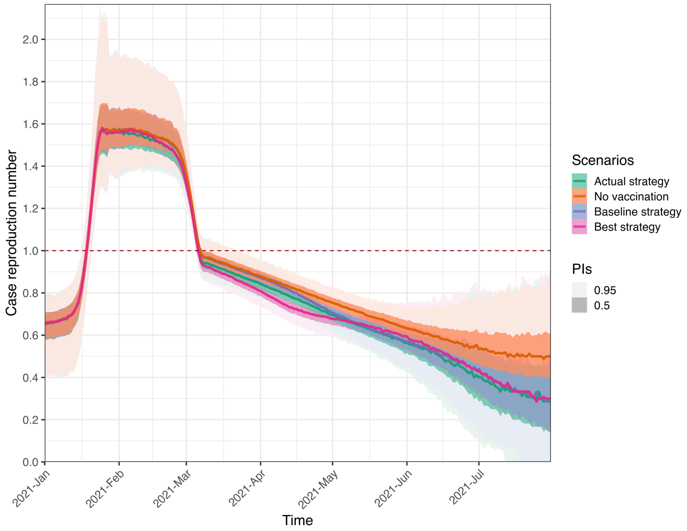

Fig S. The effective reproduction numbers (Rt) in the IBM. The case reproduction numbers from the IBM are shown given (i) the actual strategy, i.e. the reality; (ii) the scenario without vaccination; (iii) the baseline strategy without geographic prioritization; and (iv) the optimal strategy ($\Delta n = -25, \Delta p = 300\%$) for minimizing infections as shown in the main text. The case reproduction numbers are calculated by using simulated transmission chains of all infectious individuals [54–56]. The lines show the mean and the colored areas represent the 50 and 95% prediction intervals.

Table L. The age-specific probabilities of death given infection and ICU admission given hospitalization in the IBM, in the sensitivity analysis without the factor of 0.5 on the probabilities of hospitalization.

	**Probabilities of death (%)**		**Probabilities of ICU admission (%)**
**Age groups**	**Without risk factors**	**With risk factors**	**All population**
0-9	0.00009	0.0002	31.6
10-19	0.0004	0.0007	6.6
20-29	0.005	0.009	9.8
30-39	0.02	0.03	10.4
40-49	0.08	0.1	13.2
50-59	0.2	0.3	20.8
60-69	1.3	2.4	26.5
70-79	5.8	10.6	28.2
80+	19.4	35.8	13.3

We assume that the probabilities of death depend on both age and risk status while the probabilities of ICU admission depend on age only. The age-specific probabilities of death given infection are calculated based on the observed number of deaths and the estimated mean number of infections. The age-specific probabilities of ICU admission given hospitalization are calculated based on the observed numbers of ICU admissions and hospitalizations.

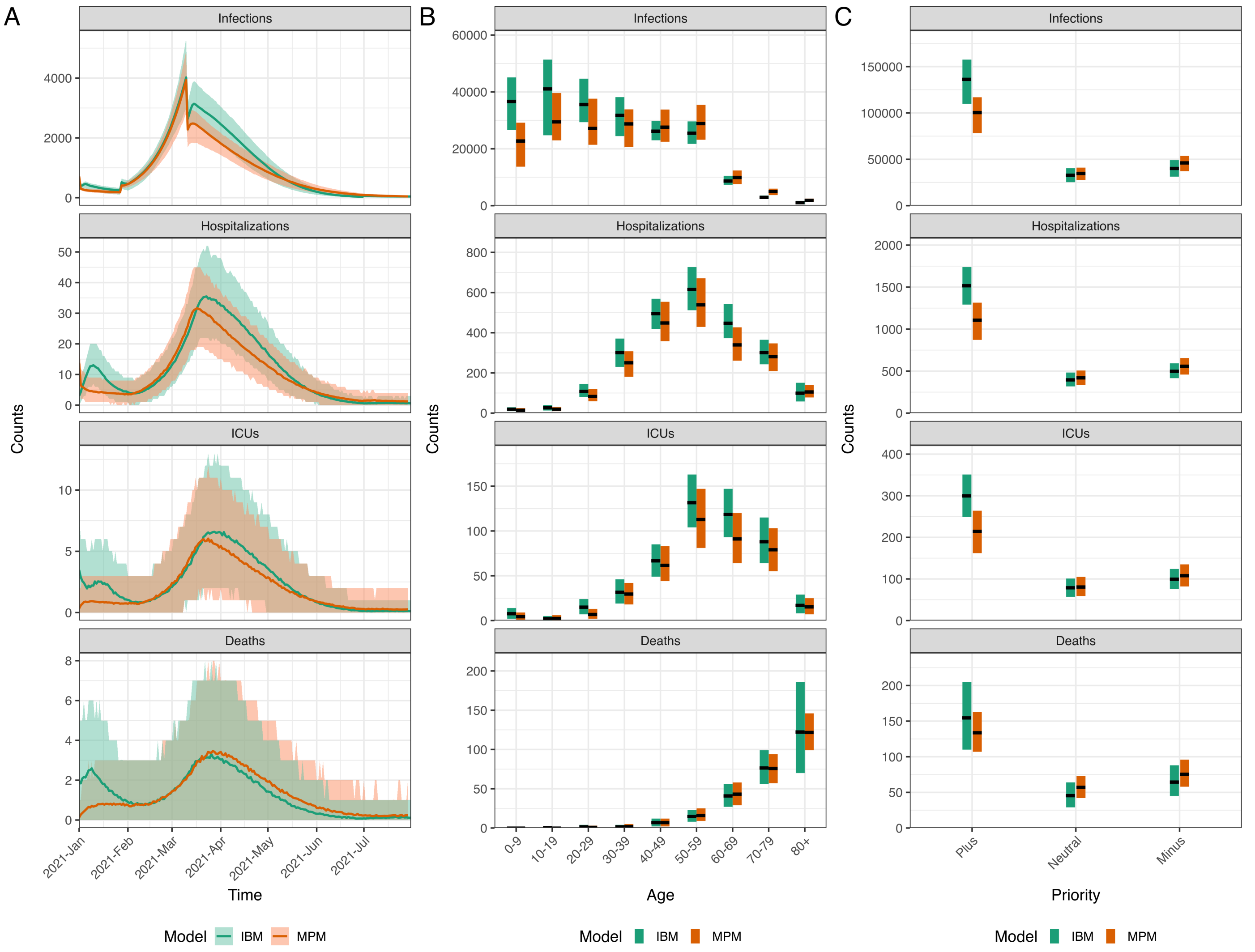

Fig T. The comparison of two models in the actual strategy. A: The time series data of all ages. The lines show the model fits with their 95% prediction intervals represented by colored areas. B: The age distribution of total counts. C: The geographic-specific priority distribution of total counts. The black lines show the median values, and the colored areas show the 95% prediction intervals.

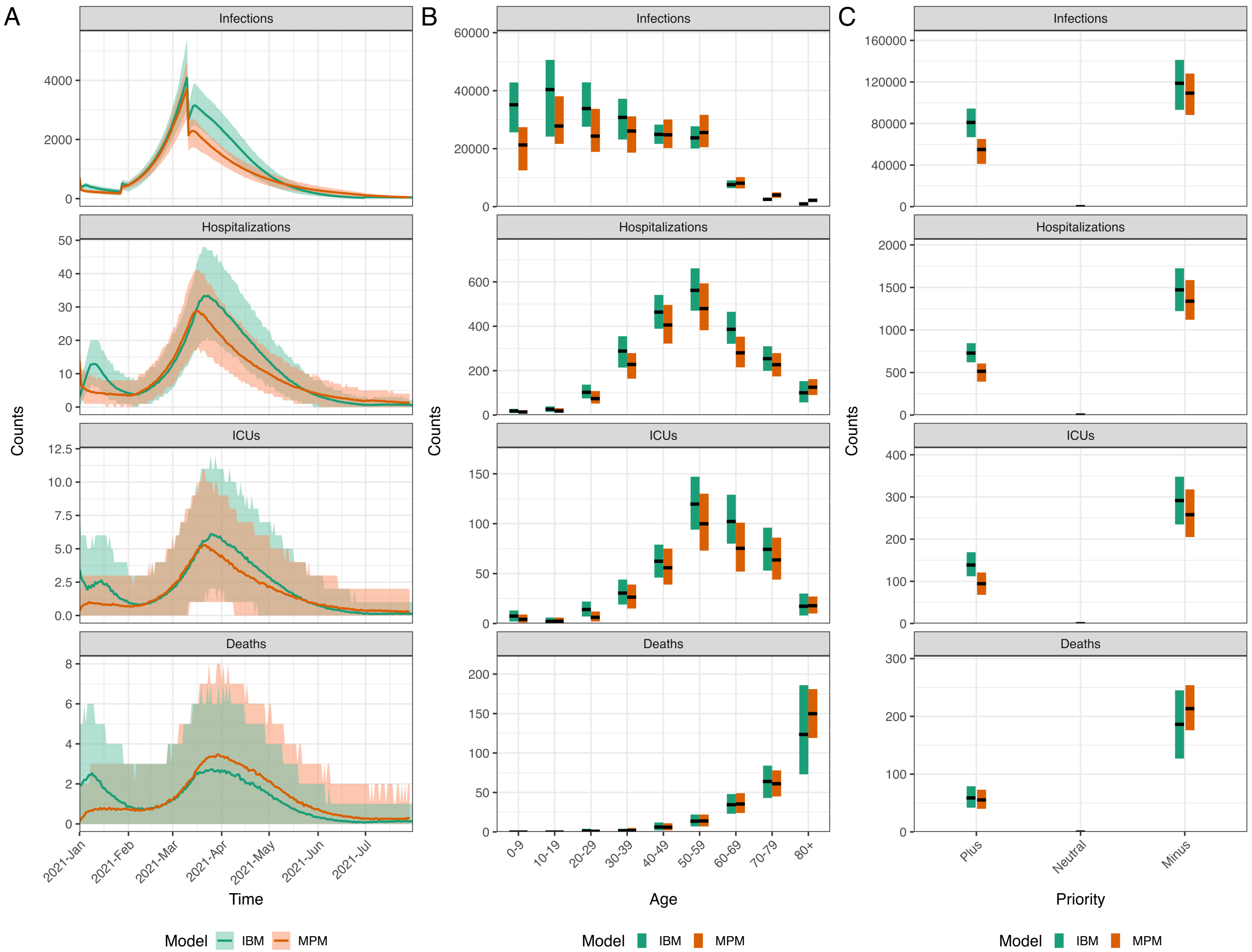

Fig U. The comparison of two models in the optimal strategy for minimizing infections. A: The time series data of all ages. The lines show the model fits with their 95% prediction intervals represented by colored areas. B: The age distribution of total counts. C: The geographic-specific priority distribution of total counts. The black lines show the median values, and the colored areas show the 95% prediction intervals.

Table M. The age-specific probabilities of death given infection and ICU admission given hospitalization in the MPM, in the sensitivity analysis without the factor of 0.5 on the probabilities of hospitalization.

	Probabilities of death (%)		Probabilities of ICU admission (%)
**Age groups**	**Without risk factors**	**With risk factors**	**All population**
0-9	0.00007	0.0001	29.2
10-19	0.0001	0.0002	13.6
20-29	0.001	0.003	8.4
30-39	0.002	0.004	11.8
40-49	0.01	0.02	13.8
50-59	0.05	0.1	21.0
60-69	0.4	0.7	27.0
70-79	2.8	5.2	28.2
80+	15.4	28.5	14.4

We assume that the probabilities of death depend on both age and risk status while the probabilities of ICU admission depend on age only. The age-specific probabilities of death given infection are calculated based on the observed number of deaths and the estimated mean number of infections. The age-specific probabilities of ICU admission given hospitalization are calculated based on the observed numbers of ICU admissions and hospitalizations.

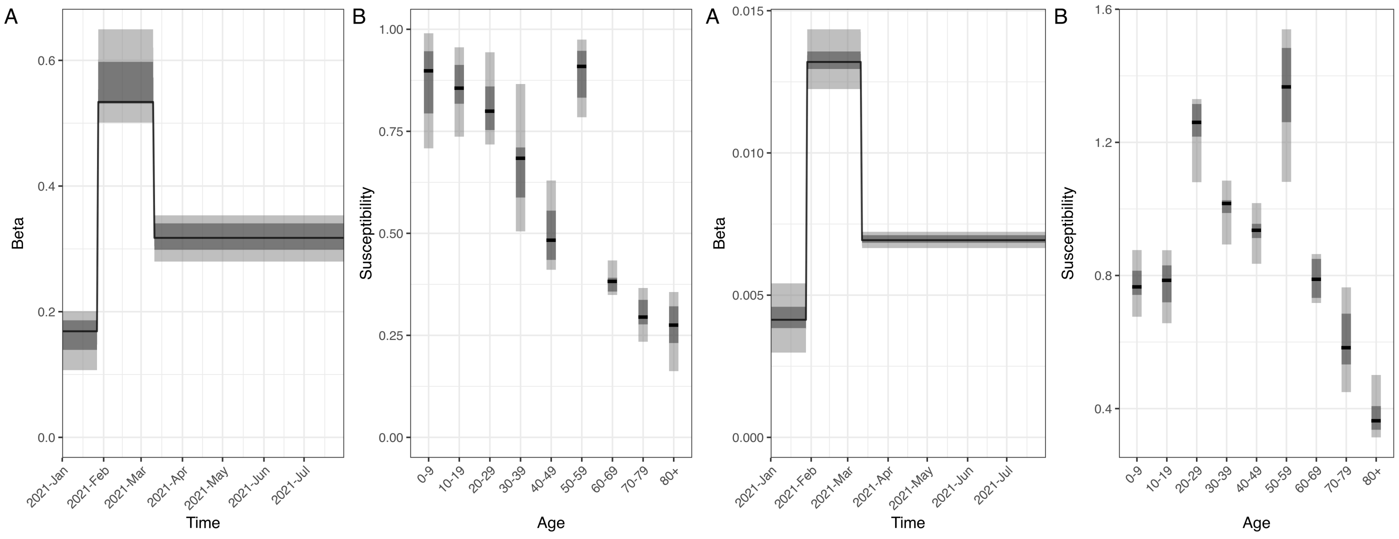

Fig V. The marginal distributions of 12 estimated parameters in both (Left) IBM and (Right) MPM given doubled hospitalization probabilities. (A) Beta in 3 time periods and (B) susceptibilities of 9 age groups are sampled from Latin hypercube sampling (LHS) approach. The black lines show the median values, and the darker (lighter) gray areas show the ranges of 25th and 75th percentiles, i.e. lower and upper quantiles (2.5th and 97.5th percentiles) of the estimated parameters.

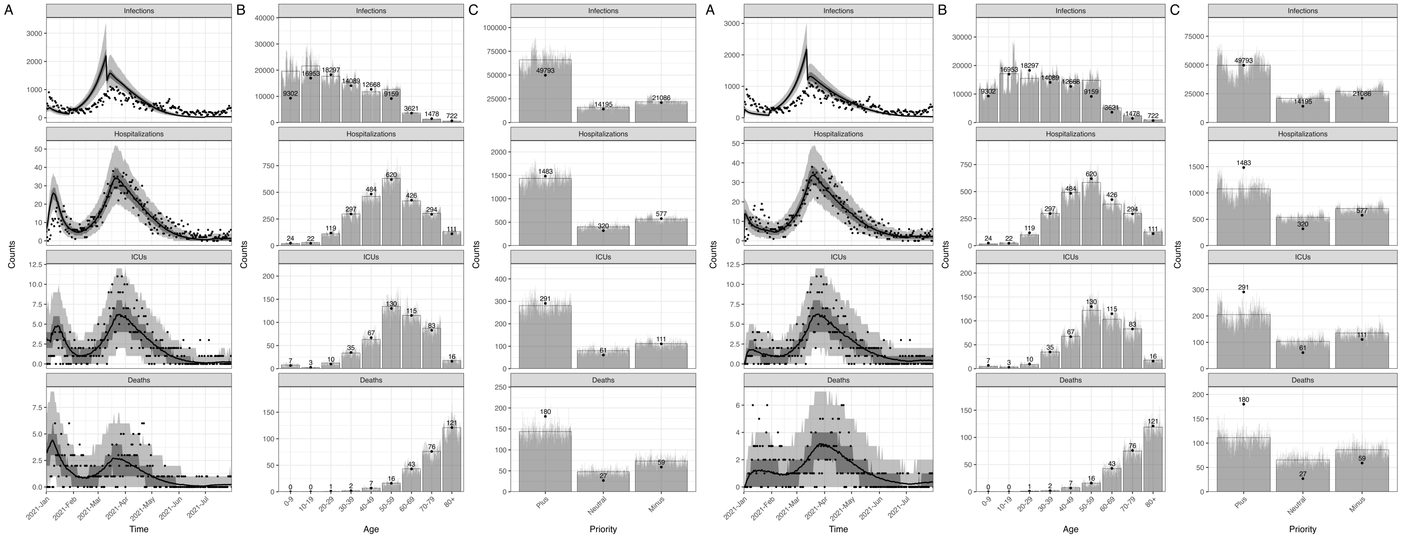

Fig W. The calibrated (Left) IBM and (Right) MPM with observed data given doubled hospitalization probabilities. A: The time series data of all ages. The lines show the model fits with their 50 and 95% prediction intervals represented by gray areas. The dots show the observed data. B: The age distribution of total counts. C: The geographic-specific priority distribution of total counts. The gray bars show the counts of each simulation and the full bars with black borders show the mean of all simulations. The data are shown in dots with their exact numbers.

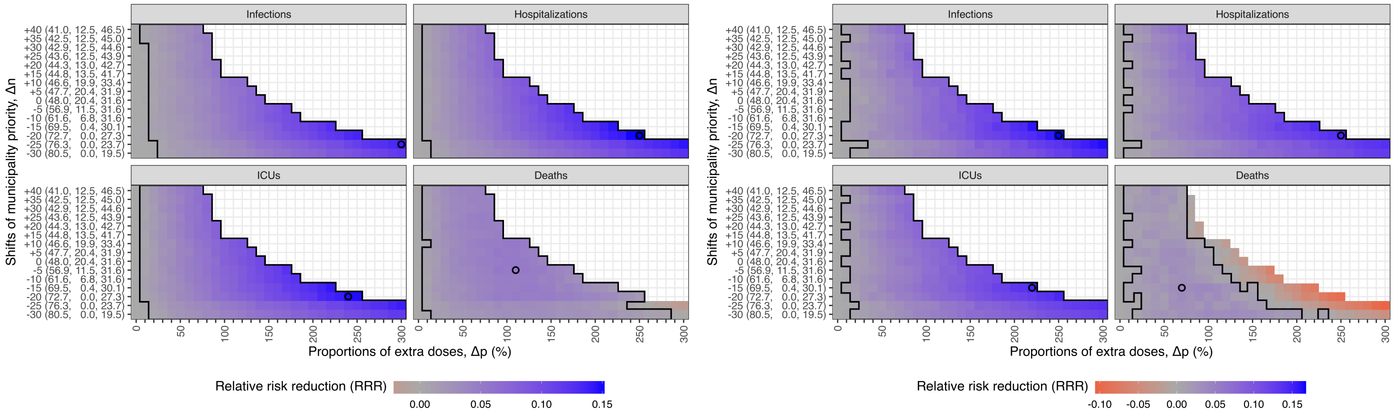

Fig X. The mean relative risk reduction (RRR) of health outcomes from both (Left) IBM and (Right) MPM in the alternative strategies given doubled hospitalization probabilities. The mean RRR of infections, hospitalizations, ICU admissions and deaths from negative to positive is represented by color from red to blue. The empty circles represent the best strategies for the highest RRR of each target. For minimizing infections, hospitalizations or ICU admissions, the higher prioritization is associated with the higher RRR. For minimizing deaths, the highest level of prioritization leads to a negative impact and medium levels of prioritization correspond to the highest RRR. The y-axis shows the population fractions (%) of three groups (*Minus*, *Neutral*, and *Plus*) for each shift in municipality priority (Δn). The geographic distribution of municipality priority (Δn) can be found in Fig 2.

Table N. The relative risk reduction (RRR) of different strategies compared to the baseline strategy without geographic prioritization, given doubled hospitalization probabilities.

		**No vaccination**	**Best strategy**
**Models**	**Outcomes**	**RRR, % (95% CI)**	**RRR, % (95% CI)** [Δp, Δn]
IBM	Infections	-4.3 (-4.8, -3.7)	13.8 (13.4, 14.2) [300%, −25]
IBM	Hospitalizations	-12.5 (-13.0, -11.9)	15.2 (14.8, 15.5) [250%, −20]
IBM	ICUs	-13.7 (-14.3, -13.0)	14.8 (14.3, 15.2) [240%, −20]
IBM	Deaths	-50.7 (-51.7, -49.7)	5.4 (5.0, 5.9) [110%, −5]
MPM	Infections	-14.3 (-14.8, -13.7)	16.8 (16.3, 17.2) [250%, −20]
MPM	Hospitalizations	-23.8 (-24.4, -23.2)	14.7 (14.3, 15.1) [250%, −20]
MPM	ICUs	-25.5 (-26.2, -24.8)	14.7 (14.2, 15.2) [220%, −15]
MPM	Deaths	-84.5 (-85.7, -83.3)	4.0 (3.3, 4.6) [70%, −15]

The mean RRR (and their 95% confidence intervals) of the scenario without vaccination and the best strategies are shown in the two columns. The best strategies, selected separately for each health outcome, are shown in square brackets.

References

1. Di Ruscio F, Guzzetta G, Bjørnholt JV, Leegaard TM, Moen AEF, Merler S, et al. Quantifying the transmission dynamics of MRSA in the community and healthcare settings in a low-prevalence country. Proceedings of the National Academy of Sciences. 2019;116(29):14599–14605. doi:10.1073/pnas.1900959116.

2. Classification of statistical tract and basic statistical unit, Statistics Norway, Norway;. Available from: https://www.ssb.no/en/klass/klassifikasjoner/1 [cited 25th January 2023].

3. Hinch R, Probert WJM, Nurtay A, Kendall M, Wymant C, Hall M, et al. OpenABM-Covid19—An agent-based model for non-pharmaceutical interventions against COVID-19 including contact tracing. PLOS Computational Biology. 2021;17(7):1–26. doi:10.1371/journal.pcbi.1009146.

4. Voigt A, Omholt S, Almaas E. Comparing the impact of vaccination strategies on the spread of COVID-19, including a novel household-targeted vaccination strategy. PLOS ONE. 2022;17(2):1–16. doi:10.1371/journal.pone.0263155.

5. Rypdal M, Rypdal V, Jakobsen PK, Ytterstad E, Løvsletten O, Klingenberg C, et al. Modelling suggests limited change in the reproduction number from reopening Norwegian kindergartens and schools during the COVID-19 pandemic. PLOS ONE. 2021;16(2):1–13. doi:10.1371/journal.pone.0238268.

6. Hoertel N, Blachier M, Blanco C, Olfson M, Massetti M, Rico MS, et al. A stochastic agent-based model of the SARS-CoV-2 epidemic in France. Nature medicine. 2020;26(9):1417–1421.

7. Chen J, Hoops S, Marathe A, Mortveit H, Lewis B, Venkatramanan S, et al. Prioritizing allocation of COVID-19 vaccines based on social contacts increases vaccination effectiveness. medRxiv. 2021;doi:10.1101/2021.02.04.21251012.

8. Goldenbogen B, Adler SO, Bodeit O, Wodke JAH, Escalera-Fanjul X, Korman A, et al. Control of COVID-19 Outbreaks under Stochastic Community Dynamics, Bimodality, or Limited Vaccination. Advanced Science. 2022;9(23):2200088. doi:https://doi.org/10.1002/advs.202200088.

9. Fumanelli L, Ajelli M, Manfredi P, Vespignani A, Merler S. Inferring the Structure of Social Contacts from Demographic Data in the Analysis of Infectious Diseases Spread. PLOS Computational Biology. 2012;8(9):1–10. doi:10.1371/journal.pcbi.1002673.

10. Statistics Norway;. Available from: https://www.ssb.no/en [cited 31st October 2022].

11. Veneti L, Robberstad B, Steens A, Forland F, Winje BA, Vestrheim DF, et al. Social contact patterns during the early COVID-19 pandemic in Norway: insights from a panel study, April to September 2020. medRxiv. 2023;doi:10.1101/2023.11.18.23298731.

12. Telenor;. Available from: https://www.telenor.com [cited 24th February 2023].

13. Ma Q, Liu J, Liu Q, Kang L, Liu R, Jing W, et al. Global Percentage of Asymptomatic SARS-CoV-2 Infections Among the Tested Population and Individuals With Confirmed COVID-19 Diagnosis: A Systematic Review and Meta-analysis. JAMA Network Open. 2021;4(12):e2137257–e2137257. doi:10.1001/jamanetworkopen.2021.37257.

14. Bubar KM, Reinholt K, Kissler SM, Lipsitch M, Cobey S, Grad YH, et al. Model-informed COVID-19 vaccine prioritization strategies by age and serostatus. Science. 2021;371(6532):916–921. doi:10.1126/science.abe6959.

15. Svar på oppdrag 8 Vaksinasjon - Delleveranse reviderte anbefalinger for geografisk prioritering, Norwegian Institute of Public Health, Norway;. Available from: https://www.fhi.no/contentassets/1af4c6e655014a738055c79b72396de8/svar-pa-oppdrag-8-vaksinasjon---delleveranse-reviderte-anbefalinger-for-geografisk-prioritering.pdf [cited 15th December 2022].

16. Modelleringsrapport, delleveranse Oppdrag 8: Effekt av regional prioritering av covid-19 vaksiner til Oslo eller Oslo- Viken samt vaksinenes effekt på transmisjon for epidemiens videre utvikling (preliminære resultater), Norwegian Institute of Public Health, Norway;. Available from: https://www.fhi.no/contentassets/1af4c6e655014a738055c79b72396de8/modelleringsrapport_delleveranse_oppdrag8_2402.pdf [cited 15th December 2022].

17. Tilleggsanalyser til modelleringsrapport, delleveranse Oppdrag 8: Effekt av moderat regional prioritering av covid-19 vaksiner til utvalgte kommuner – 5. Mars 2021, Norwegian Institute of Public Health, Norway;. Available from: https://www.fhi.no/contentassets/1af4c6e655014a738055c79b72396de8/vedlegg-5-til-delleveranse-8.pdf [cited 15th December 2022].

18. Modelleringsrapport til Oppdrag 8, Norwegian Institute of Public Health, Norway;. Available from: https://www.fhi.no/contentassets/e6b5660fc35740c8bb2a32bfe0cc45d1/vedlegg/nasjonale-og-regionale-rapporter/oppdrag_8_2303_bfdblasio.pdf [cited 15th December 2022].

19. Dagan N, Barda N, Kepten E, Miron O, Perchik S, Katz MA, et al. BNT162b2 mRNA Covid-19 Vaccine in a Nationwide Mass Vaccination Setting. New England Journal of Medicine. 2021;384(15):1412–1423. doi:10.1056/NEJMoa2101765.

20. Davies NG, Klepac P, Liu Y, Prem K, Jit M, Eggo RM. Age-dependent effects in the transmission and control of COVID-19 epidemics. Nature medicine. 2020;26(8):1205–1211.

21. Rozhnova G, van Dorp CH, Bruijning-Verhagen P, Bootsma MC, van de Wijgert JH, Bonten MJ, et al. Model-based evaluation of school-and non-school-related measures to control the COVID-19 pandemic. Nature communications. 2021;12(1):1–11.

22. Viana J, van Dorp CH, Nunes A, Gomes MC, van Boven M, Kretzschmar ME, et al. Controlling the pandemic during the SARS-CoV-2 vaccination rollout. Nature communications. 2021;12(1):1–15.

23. Jing QL, Liu MJ, Zhang ZB, Fang LQ, Yuan J, Zhang AR, et al. Household secondary attack rate of COVID-19 and associated determinants in Guangzhou, China: a retrospective cohort study. The Lancet Infectious Diseases. 2020;20(10):1141–1150. doi:https://doi.org/10.1016/S1473-3099(20)30471-0.

24. Goldstein E, Lipsitch M, Cevik M. On the Effect of Age on the Transmission of SARS-CoV-2 in Households, Schools, and the Community. The Journal of Infectious Diseases. 2020;223(3):362–369. doi:10.1093/infdis/jiaa691.

25. Veneti L, Seppälä E, Larsdatter Storm M, Valcarcel Salamanca B, Alnes Buanes E, Aasand N, et al. Increased risk of hospitalisation and intensive care admission associated with reported cases of SARS-CoV-2 variants B.1.1.7 and B.1.351 in Norway, December 2020 –May 2021. PLOS ONE. 2021;16(10):1–12. doi:10.1371/journal.pone.0258513.

26. Andrews N, Tessier E, Stowe J, Gower C, Kirsebom F, Simmons R, et al. Duration of Protection against Mild and Severe Disease by Covid-19 Vaccines. New England Journal of Medicine. 2022;386(4):340–350. doi:10.1056/NEJMoa2115481.

27. Risiko ved covid-19- epidemien og ved omikronvarianten i Norge, Norwegian Institute of Public Health, Norway;. Available from: https://www.fhi.no/contentassets/c9e459cd7cc24991810a0d28d7803bd0/vedlegg/risikovurdering-2021-12-13.pdf [cited 19th April 2023].

28. Modelling scenarios for the SARS-CoV-2 Omicron VOC (B.1.1.529) in Norway during the winter 2021—2022, Norwegian Institute of Public Health, Norway;. Available from: https://www.fhi.no/contentassets/e6b5660fc35740c8bb2a32bfe0cc45d1/vedlegg/nasjonale-og-regionale-rapporter/omicron_modelling_report_2021_12_22.pdf [cited 19th April 2023].

29. Modelling scenarios for the SARS-CoV-2 Omicron VOC (B.1.1.529) in Norway, January-February 2022, Norwegian Institute of Public Health, Norway;. Available from: https://www.fhi.no/contentassets/e6b5660fc35740c8bb2a32bfe0cc45d1/vedlegg/nasjonale-og-regionale-rapporter/modelling-scenarios-for-the-sars-cov-2-omicron-voc-b.1.1.529-in-norway-january-february-2022.pdf [cited 19th April 2023].

30. Modelling scenarios for the SARS-CoV-2 Omicron VOC (B.1.1.529) in Norway, gradual reopening in February-March 2022, Norwegian Institute of Public Health, Norway;. Available from: https://www.fhi.no/contentassets/e6b5660fc35740c8bb2a32bfe0cc45d1/vedlegg/nasjonale-og-regionale-rapporter/modelling-scenarios-for-the-sars-cov-2-omicron-voc-26.01.2022.pdf [cited 19th April 2023].

31. Ferretti L, Wymant C, Kendall M, Zhao L, Nurtay A, Abeler-Dörner L, et al. Quantifying SARS-CoV-2 transmission suggests epidemic control with digital contact tracing. Science. 2020;368(6491):eabb6936. doi:10.1126/science.abb6936.

32. Situational awareness and forecasting for Norway on 20th January 2021, Norwegian Institute of Public Health, Norway;. Available from: https://www.fhi.no/contentassets/e6b5660fc35740c8bb2a32bfe0cc45d1/vedlegg/nasjonale-og-regionale-rapporter/2021.01.20-national-and-regional-corona-report-engelsk.pdf [cited 12th December 2022].

33. Coronavirus modelling at the NIPH, Norwegian Institute of Public Health, Norway;. Available from: https://www.fhi.no/en/id/infectious-diseases/coronavirus/coronavirus-modelling-at-the-niph-fhi/ [cited 19th October 2021].

34. Engebretsen S, Diz-Lois Palomares A, Rø G, Kristoffersen AB, Lindstrøm JC, Engø-Monsen K, et al. A real-time regional model for COVID-19: Probabilistic situational awareness and forecasting. PLOS Computational Biology. 2023;19(1):1–26. doi:10.1371/journal.pcbi.1010860.

35. Carpenter B, Gelman A, Hoffman MD, Lee D, Goodrich B, Betancourt M, et al. Stan: A probabilistic programming language. Journal of statistical software. 2017;76(1).

36. Stan Development Team. RStan: the R interface to Stan; 2022. Available from: http://mc-stan.org/.

37. Herrera-Esposito D, de Los Campos G. Age-specific rate of severe and critical SARS-CoV-2 infections estimated with multi-country seroprevalence studies. BMC infectious diseases. 2022;22(1):1–14.

38. Grint DJ, Wing K, Houlihan C, Gibbs HP, Evans SJW, Williamson E, et al. Severity of Severe Acute Respiratory System Coronavirus 2 (SARS-CoV-2) Alpha Variant (B.1.1.7) in England. Clinical Infectious Diseases. 2021;75(1):e1120–e1127. doi:10.1093/cid/ciab754.

39. Pascall DJ, Vink E, Blacow R, Bulteel N, Campbell A, Campbell R, et al. The SARS-CoV-2 Alpha variant is associated with increased clinical severity of COVID-19 in Scotland: a genomics-based retrospective cohort analysis. MedRxiv. 2022; p. 2021–08.

40. Bager P, Wohlfahrt J, Fonager J, Rasmussen M, Albertsen M, Michaelsen TY, et al. Risk of hospitalisation associated with infection with SARS-CoV-2 lineage B.1.1.7 in Denmark: an observational cohort study. The Lancet Infectious Diseases. 2021;21(11):1507–1517. doi:https://doi.org/10.1016/S1473-3099(21)00290-5.

41. Funk T, Pharris A, Spiteri G, Bundle N, Melidou A, Carr M, et al. Characteristics of SARS-CoV-2 variants of concern B.1.1.7, B.1.351 or P.1: data from seven EU/EEA countries, weeks 38/2020 to 10/2021. Eurosurveillance. 2021;26(16). doi:https://doi.org/10.2807/1560-7917.ES.2021.26.16.2100348.

42. Tunheim G, Rø G[U+FFFD]I, Tran T, Kran AMB, Andersen JT, Vaage EB, et al. Trends in seroprevalence of SARS-CoV-2 and infection fatality rate in the Norwegian population through the first year of the COVID-19 pandemic. Influenza and Other Respiratory Viruses. 2022;16(2):204–212. doi:https://doi.org/10.1111/irv.12932.

43. Seroprevalence of SARS-CoV-2 in the Norwegian population measured in residual sera collected in January 2021, Norwegian Institute of Public Health, Norway;. Available from: https://www.fhi.no/en/publ/2021/seroprevalence-of-sars-cov-2-in-the-norwegian-population-measured-in-residu/ [cited 10th October 2022].

44. Seroprevalence of SARS-CoV-2 in the Norwegian population measured in residual sera collected in April/May 2020 and August 2019, Norwegian Institute of Public Health, Norway;. Available from: https://www.fhi.no/en/publ/2020/seroprevalence-of-sars-cov-2-in-the-norwegian-population--measured-in-resid/ [cited 10th October 2022].

45. Anda EE, Braaten T, Borch KB, Nøst TH, Chen SL, Lukic M, et al. Seroprevalence of antibodies against SARS-CoV-2 virus in the adult Norwegian population, winter 2020/2021: pre-vaccination period. medRxiv. 2021;.

46. Emergency preparedness register for COVID-19 (Beredt C19), Norwegian Institute of Public Health, Norway;. Available from: https://www.fhi.no/en/id/infectious-diseases/coronavirus/emergency-preparedness-register-for-covid-19/ [cited 24th January 2023].

47. Folkehelseinstituttets foreløpige anbefalinger om vaksinasjon mot covid-19 og om prioritering av covid-19-vaksiner, versjon 2, Norwegian Institute of Public Health, Norway;. Available from: https://www.fhi.no/contentassets/d07db6f2c8f74fa586e2d2a4ab24dfdf/2020-12-v2-anbefalinger-og-prioriteringer-2-utgave-korrigert-forside.pdf [cited 15th December 2022].

48. Norwegian Surveillance System for Communicable Diseases (MSIS), Norwegian Institute of Public Health, Norway;. Available from: https://www.fhi.no/en/hn/health-registries/msis/ [cited 31st October 2022].

49. Norsk pandemiregister, Helse Bergen, Norway;. Available from: https://helse-bergen.no/norsk-pandemiregister [cited 2nd February 2023].

50. Norwegian Intensive Care and Pandemic Registry (NIPaR), Helsedata, Norway;. Available from: https://helsedata.no/en/forvaltere/bergen-hospital-trust/norwegian-intensive-care-and-pandemic-registry/ [cited 2nd February 2023].

51. Norwegian Immunisation Registry SYSVAK, Norwegian Institute of Public Health, Norway;. Available from: https://www.fhi.no/en/hn/health-registries/norwegian-immunisation-registry-sysvak/ [cited 19th October 2021].

52. Regjeringen har besluttet en geografisk omfordeling av vaksiner, Helse- og omsorgsdepartementet, Norway;. Available from: https://www.regjeringen.no/no/aktuelt/regjeringen-har-besluttet-en-geografisk-omfordeling-av-vaksiner/id2850061/ [cited 19th October 2021].

53. Nye vurderinger av vaksinasjonsstrategien, Norwegian Institute of Public Health, Norway;. Available from: https://www.fhi.no/contentassets/3596efb4a1064c9f9c7c9e3f68ec481f/svar-pa-oppdrag-16---nye-vurderinger-av-vaksinasjonsstrategien-inkl.-vedlegg_skjult-innhold.pdf [cited 15th December 2022].

54. Cori A, Ferguson NM, Fraser C, Cauchemez S. A New Framework and Software to Estimate Time-Varying Reproduction Numbers During Epidemics. American Journal of Epidemiology. 2013;178(9):1505–1512. doi:10.1093/aje/kwt133.

55. Fraser C. Estimating Individual and Household Reproduction Numbers in an Emerging Epidemic. PLOS ONE. 2007;2(8):1–12. doi:10.1371/journal.pone.0000758.

56. Wallinga J, Teunis P. Different Epidemic Curves for Severe Acute Respiratory Syndrome Reveal Similar Impacts of Control Measures. American Journal of Epidemiology. 2004;160(6):509–516. doi:10.1093/aje/kwh255.
