# Supplementary material for: Modeling geographic vaccination strategies for COVID-19 in Norway
Source: PLoS Comput Biol. 2024 Jan 31;20(1):e1011426. doi: 10.1371/journal.pcbi.1011426 (PMC10861074; doi:10.1371/journal.pcbi.1011426)
Supplement: Attachment — Submitted filename: Response_letter.pdf [file pcbi.1011426.s002.pdf]

Dear editors,

We would like to express our gratitude for the thorough review of our manuscript titled "Modeling Geographic Vaccination Strategies for COVID-19 in Norway". We appreciate the valuable feedback provided by the reviewers, which has greatly contributed to the improvement of our work.

In response to the insightful comments, we have diligently revised the manuscript to address each concern and enhance the overall quality of our research. Below is a detailed summary of the review comments and the corresponding changes made.

Thank you for the opportunity to revise and resubmit our manuscript. We look forward to your feedback and appreciate your time and consideration.

Sincerely,
Louis Yat Hin Chan
Department of Method Development and Analytics, Norwegian Institute of Public Health, Oslo, Norway
imlouischan@gmail.com

Reviewer #1:
The authors provide a clear and well written account of the modeling efforts conducted by the Norwegian Institute of Public Health to evaluate different allocation strategies for COVID-19 vaccines and guide policy making. Methodology is well grounded, with good integration between modeling efforts and calibration using good quality data, the sources of which are (almost) all clearly documented.

> **Response: We appreciate the reviewer's feedback and have made revisions. Please find below our point-to-point responses.**

I only have two big concerns regarding the work (outlined in points 3 and 4 below), but I believe they should not impede publication as long as they are properly addressed. Points 1 and 2 outline minor improvements.

1) I commend the authors for the good quality of the text and figures, and my only request in this front is to please use the appropriate command for starting quotations: the first mark is flipped as in line 202 and in the Acknowledgments section; please check if I missed other uses. It also would be nice (but not essential) if Fig 1 followed the color scheme for ABM used in Fig 6, since all the other figures are tastefully colored.

- **We have corrected the two quotations.**

> We vaccinate individuals who are not infected at the time of vaccination and assume that the vaccines are ~~”leaky”~~ “leaky”, meaning that the vaccine effect of both the first

> ## 8 Acknowledgments
>
> The project was funded by the Research Council of Norway ~~”~~ “COVID-19 in Norway: A real-time analytical pipeline for preparedness, planning and response during the COVID-19 pandemic in Norway~~”~~” grant number 312721. The mobility data was collected and provided by Telenor Norway.

- **We have updated Fig 1 with the same colors as Fig 6.**

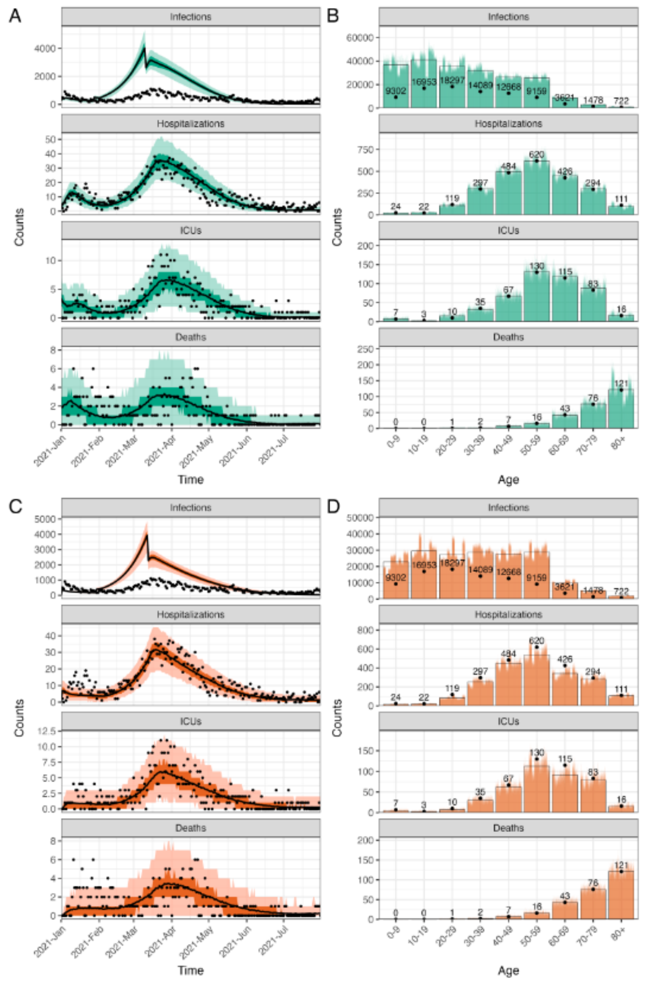


**Fig 1. The calibrated individual-based model (IBM) and meta-population model (MPM) with data in the actual vaccination strategy.** The upper and lower panels show the IBM and MPM, respectively. A and C: The time series data of all ages. The lines show the model fits with their 50 and 95% prediction intervals represented by ~~gray~~ colored areas based on 1000 simulations. The dots show the observed data. B and D: The age distribution of total counts. ~~The gray~~ Each of the 1000 colored bars ~~show~~ shows the counts ~~of each~~ from one simulation and the full bars with black borders show the mean of all simulations. The data are shown in dots with their exact numbers. During the whole period, there were 86289 confirmed cases, 2397 hospital admissions, 466 ICU admissions, and 266 deaths registered.

2) The social settings for contacts between agents need to be clarified in two instances:

2.1) The "Community setting" for contacts is not properly defined as it is first mentioned;

- **We have added a sentence to define “community” while it is first mentioned in Section 4.2.1.**

> The community contacts refer to interactions that take place outside of the household, school, university, or workplace, and may include activities such as taking public transportation, grocery shopping, and socializing with friends. These contacts are modeled as random processes ~~based on~~ following a negative binomial distribution to represent super-spreading events [45] and taking into consideration the age of potential contacts and physical distance between cells. Relative contact rates among different age groups are ~~also considered, with the highest contact rate observed among those aged 20 to 29 years according to the unpublished social contact studies~~taken into account based on the social contact study survey [11]. To reflect real-world mobility patterns, we employ a heavy-tailed distribution function based on mobility data from the largest telecommunication company in Norway, Telenor [12, 34]. This function results in a greater likelihood of short-distance transmission compared to long-distance transmission.

2.2) In paragraph between lines 287 and 291, please cite source and give a brief account of the methodology that led to the proportions of infections in each of the settings explored. Moreover, the Community social setting is not listed here and you only mention "other settings"; are these "other settings" the definition for the Community setting that was missing as it was first mentioned?

- **We have added a citation showing that the data were collected by Norwegian Surveillance System for Communicable Diseases (MSIS).**

> The IBM was additionally calibrated to fit the proportions of the four transmission routes based on information provided by general practitioners regarding likely transmissions and collected by Norwegian Surveillance System for Communicable Diseases (MSIS) [37]. The data ~~collected from general practitioners about the likely transmission~~ revealed that 45.1%, ~~9.9%, 6.2~~38.6%, 9.9%, and ~~38.6~~6.2% of cases from January to July 2021 were infected within households, ~~workplaces, schools, and other settings, respectively.~~ community, workplaces, and schools, respectively. We used these proportions to establish specific weights for scaling the transmission rate in each setting.

- **We have replaced the “other settings” by “community” to be consistent throughout the entire article.**

> January to July 2021 were infected within households, ~~workplaces, schools, and other settings,~~ community, workplaces, and schools, respectively.

3) The authors mention that contacts are modeled based on an unpublished social contact studies (105-106).

3.1) Later on, they explain that contact are uniformly distributed for agents within household, schools, universities and workplaces, which leaves only the Community setting (which was already vague) to follow any heterogeneous contact structure. I therefore find the statements in lines (105-106) misleading as it implies data integration of observed contact patterns to a greater degree than actually performed. Again, in lines 506 and 507, the authors mention that heterogeneous contact structure was employed in all four contact settings; this clashes with their explanation that contacts were uniformly distributed in the This needs to be addressed!

- **We have added the explanation of contacts within households, schools, universities, and workplaces in Section 4.2.1.**

> Contacts within specific households, schools, universities, and workplaces are ~~uniformly distributed among all individuals belonging to those institutions. The IBM's structure~~ assumed to mix randomly. Individuals are assigned to specific households and establishments based on age-structured census data, with middle-aged individuals more likely to reside with their spouse and children, and the elderly often living in pairs or alone. Younger individuals are assigned to schools, while adults are more likely to be in workplaces. Hence, contacts within households and occupations are assumed age-dependent without using an explicit contact matrix, as in the case of community contacts. The IBM was originally developed to study the transmission dynamics of MRSA in Norway [1] and has been adapted to model COVID-19 transmission, as presented in this paper. A main difference is that the current version does not include hospital settings, and healthcare workers are assumed to have the same contact patterns as other occupational groups.

3.2) Contact structured is a fundamental element of ABM, and therefore cannot be obscured. Please, address
- if there are valid reasons for this data to remain unpublished;
- if it will be published soon and give enough information for us and the readers to be able to find it later on;
- or include reasonable summary of these studies and their results in the SI (if it was already done and I missed it, please cite the appropriate section of the SI in your original lines).

- **We have added the citation. The social contact study was recently published on medRxiv [https://doi.org/10.1101/2023.11.18.23298731].**

> Appendix. Moreover, we rely on ~~unpublished social contact studies~~ a social contact study survey conducted in 2017 [43] to determine the heterogeneity of contact patterns

4) The approach to contact modeling was quite limiting, mainly because
4.1.1) if does not account for heterogeneities in the contact venues employed (different types of workplaces can have very different contact structures with terrible consequences for disease propagation like in accounts of meat processing plants in various countries);

- **We did not model heterogeneous contact patterns in "workplaces" in the ABM. This was due to the lack of available data that could inform such a level of granularity. We acknowledge this limitation, recognizing that our model assumes the same pattern for all types of interactions among colleagues.**

4.1.2) it does not account for multiplicity of roles of a contact venue; for example: where does a restaurant or supermarket place in your venue classification? It is certainly a very relevant social setting and their workers can be effective super spreaders, but at the same time this is not a "workplace" for their customers, so are they not meeting with the workers in your contact model? Then, how could the model hope to represent super-spreading events in these and any other setting? And If so, fundamental mechanism of social interaction which are very important for disease propagation and play a large role in indirect protection conferred by good

vaccination policies are not being captured at all and the true potential of ABMs is not being realized.

- **In the ABM, interactions in "workplaces" pertain to transmissions between colleagues, constituting a smaller fraction, about one-tenth of all transmissions.**
- **On the other hand, interactions such as those between customers and workers are categorized as "community", as transmissions occur during activities like grocery shopping. More than one-third of all transmissions occur in these community interactions.**
- **We assumed that the number of contacts in the "community" follows a negative binomial distribution, representing potential super-spreading events. We have added this in Section 4.2.1.**

> transportation, grocery shopping, and socializing with friends. These contacts are modeled as random processes ~~based on~~ following a negative binomial distribution to represent super-spreading events [45] and taking into consideration the age of

4.1.3) the assignment of roles to agents (e.g. front line workers) in the ABM model is not described in its appropriated place (section 4.2.1) and only assumed in the Results section. How are these roles assigned, and are any other types of roles assigned to the agents? How do the roles affect contacts?

- **In the ABM, we do not assign higher contact rates to healthcare workers; rather, we assume that individuals in these roles share the same contact patterns as others. Our model only prioritizes vaccination as a primary focus. We have added our assumption in Section 4.2.1.**

> Contacts within specific households, schools, universities, and workplaces are ~~uniformly distributed among all individuals belonging to those institutions. The IBM's structure~~ assumed to mix randomly. Individuals are assigned to specific households and establishments based on age-structured census data, with middle-aged individuals more likely to reside with their spouse and children, and the elderly often living in pairs or alone. Younger individuals are assigned to schools, while adults are more likely to be in workplaces. Hence, contacts within households and occupations are assumed age-dependent without using an explicit contact matrix, as in the case of community contacts. The IBM was originally developed to study the transmission dynamics of MRSA in Norway [1] and has been adapted to model COVID-19 transmission, as presented in this paper. A main difference is that the current version does not include hospital settings, and healthcare workers are assumed to have the same contact patterns as other occupational groups.

4.2) I do not hope for a rework of the models to improve contact mechanics, and I believe that your primary intent of documenting the efforts for policy design during that pandemic needs to be respected. However, I would expect improvement in the discussion of the limitations of the contact model employed. These limitations and others you already mentioned are shared by most models and this is a point that the community needs to be better informed.

- **We have added this in Limitations.**

> rates as the general population in our models. In the IBM, we assumed a common contact structure for work-related interactions based on census data. Consequently, we did not distinguish healthcare workers and individuals employed in restaurants or supermarkets from other occupational groups. Incorporating super-spreading events was confined to community transmission, modeled by adherence to a negative binomial distribution [45].
>
> [illegible]

Despite these concerns, this is a very good work. I find that the project's methodology is very sober and responsible: the hypothesis made are clear, fit reasonably with the designed methodology and provide coherent and valuable guides for policymakers. I congratulate the authors for the work and hope they appreciate the points mentioned in a positive light, recommending this manuscript for publication after these improvements.

- **Thank you very much for the comments.**

Reviewer #2:

The authors study the effect of geographic prioritization of vaccination on the reduction of COVID-19 infections and severe outcomes , using two models. Their study provides valuable guidance for policy makers involved in making decisions about vaccination strategies. The paper is well written and the models are described in detail. I only have a few minor comments that need to be addressed before I can recommend the paper for publication.

> **Response: We appreciate the reviewer's feedback and have made revisions. Please find below our point-to-point responses.**

• Line 392: The following results shows -> … show

- **We have updated it.**

> outcomes, respectively, compared to the baseline strategy. The following results ~~shows~~ show the mean values and their 95% confidence intervals (95%CIs) using $\pm 1.96\frac{\sigma}{\sqrt{N}}$,

• Line 393: 95% confidence intervals for 1000 simulations. Can be only used if the distribution is (close to) symmetric/normal. In case of a skewed distribution, it's better to report median and IQR. Did you check whether the distribution was symmetric or skewed?

Same question for other parts of the paper where a 95% confidence interval was used.

- **We have confirmed the symmetry and normal distribution of RRR by visually verifying it through Q-Q (quantile-quantile) plots, where the data points align closely with a straight line.**

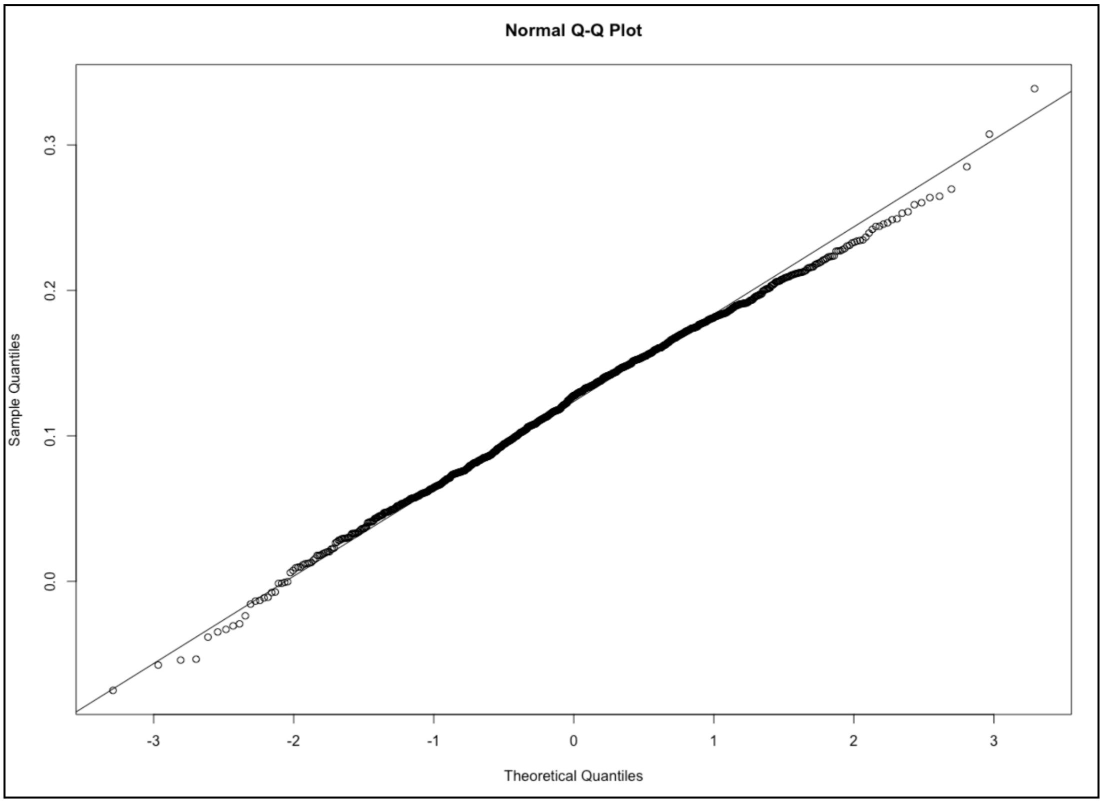

- S1.1.4. How did you obtain the percentages 40% asymptomatic / 60% pre-symptomatic?
    - **We have added a sentence with a citation. The percentage is the assumption throughout our analyses during the pandemic, and the number aligns with a systematic review [http://dx.doi.org/10.1001/jamanetworkopen.2021.37257].**

> symptomatic is 60%. This is the assumption throughout our analyses during the pandemic, and the 40% asymptomatic fraction aligns with findings from a systematic review [13]. Symptomatic individuals may either recover or die, as shown

- S1.2.2. of of travelers -> of travelers
    - **We have updated it.**

> **S1.2.2 Regional contact matrix**
>
> The regional contact matrix is based on mobility data from Telenor [12] aggregated to the number of movements between the specified regions. We define the contact matrix such that amount of infectiousness that ~~"leaks"~~ "leaks" out of a region is equal to the fraction of the population who traveled out of the region in one day. If the number ~~of~~ of travelers from region $i$ to region $j$ is $T_{ij}$ and the population in region $i$ is $P_i$, then the regional contact matrix is given by:

Reviewer #3:
This paper is interesting since it presents a study of vaccine allocation based on geographic strategies, which is important regarding large countries with heterogeneous densities. Several previous studies during covid-19 times have been carried out concerning prioritisation according to age in relation to different targets (transmission/deaths/etc.) but here the publication of a method to study geographical allocation of vaccine is quite a novelty. The two presented models are quite complex and robust and should be able to well capture the presented elements. The paper is very well written, with the main article containing (almost) all the important information for a wider audience, and a very complete supplementary material describing in details the two models, the assumptions, the methodology and the detailed results. For these reasons, I recommend its publication in PLOS Computational Biology.

> **Response: We appreciate the reviewer's feedback and have made revisions. Please find below our point-to-point responses.**

Here are some minor comments, sometimes regarding items presented in the supplementary material that could at least be mentioned in the main document for clarity:
- L105: Little additional information should already be given here regarding the origin of the social contract matrix (before the pandemic, 2017 in Norway, only mentioned in the limitations and supplementary materials)

- **We have added the citation. The social contact study was recently published on medRxiv [https://doi.org/10.1101/2023.11.18.23298731].**

Appendix. Moreover, we rely on ~~unpublished social contact studies~~ a social contact study survey conducted in 2017 [43] to determine the heterogeneity of contact patterns

- L284/Fig1caption/etc.: It's is not specified in the main document how the uncertainty is generated, especially concerning estimated parameters. In the supplementary material, there is the mention of selecting the best 10 sets of parameters of a best fit method, which should be mentioned somewhere in the main text. Are the 95% confidence intervals based on those 10 sets (not so logical) ? Is it with additional stochastic realisations ?

- **The uncertainty is based on a total of 1000 simulations, which are generated by 100 stochastic seeds for each of the best 10 sets. In Section 4.7, we wrote “We generated 100 realizations corresponding to the 100 stochastic seeds for each of 10 calibrated parameter sets. ”.**
- **In Figures 1 and 6, we have updated the captions.**

**Fig 1. The calibrated individual-based model (IBM) and meta-population model (MPM) with data in the actual vaccination strategy.** The upper and lower panels show the IBM and MPM, respectively. A and C: The time series data of all ages. The lines show the model fits with their 50 and 95% prediction intervals represented by ~~gray~~ colored areas based on 1000 simulations. The dots show the observed data. B and D: The age distribution of total counts. ~~The gray~~ Each of the 1000 colored bars ~~show~~ shows the counts ~~of each~~ from one simulation and the full bars with black borders show the mean of all simulations. The data are shown in dots with their exact numbers. During the whole period, there were 86289 confirmed cases, 2397 hospital admissions, 466 ICU admissions, and 266 deaths registered.

**Fig 6. The comparison of two models for the optimal strategy for minimizing infections.** A: The time series data of all ages. The lines show the model fits with their 95% prediction intervals represented by colored areas based on 1000 simulations. B: The age distribution of total counts. The black lines show the median values, and the colored areas show the 95% prediction intervals based on 1000 simulations.

- Table 1/Fig3/L558: There is a clear difference concerning optimal strategies with deaths target between the two models, with only a very short comment "likely due to slightly different population compositions" in the discussion section. Might be good to have a broader analysis here. What could be the elements that could be responsible for this difference? calibration? internal model differences? other things?

- **We have added a part to explain the differences in Discussion.**

### 6.3 Deaths

To minimize deaths, an early and geographically extended prioritization encompassing 21-34 municipalities comprising approximately 30% of the Norwegian population (alongside approximately 10 *Neutral* municipalities representing approximately 20% of the Norwegian population) and a more moderate level of priority, approximately doubling their vaccine doses, yielded the most favorable outcomes. The optimal strategies ~~differed somewhat~~ slightly differed between the two models, ~~likely due to slightly different population compositions . The~~ due to variations in population compositions and the geographic breakdown of the country. The IBM modeled all 356 municipalities, one-year age groups and different settings of transmission, while the MPM was limited to a maximum of 15 geographic regions and 9 ten-year age groups. These distinctions also affect the initial conditions and calibration process, resulting in different outcomes in the two models. Due to the exponential increase in death probabilities with age, even small changes in the elderly population led to noticeable differences in the resulting strategies. The results highlight the importance of determining the primary purpose of the vaccination program. In Norway, the government appointed an ethics committee that concluded a vaccination program aimed at minimizing the number of deaths should be chosen [62]. As we demonstrate, this leads to a significantly different optimal strategy than if the objective were to reduce hospitalizations, for instance. Moreover, we anticipate that minimizing disability-adjusted or quality-adjusted life year lost would also necessitate more geographic prioritization, rather than solely focusing on deaths [63].

- Conclusion and Discussion section: This section is one continuous block of text that is far too long. It would be more readable if it were divided into thematic subsections.

- **We have split it into 8 subsections.**

Conclusion and Discussion
- Merit of this study
- Infections, hospitalizations, and ICU admissions
- Deaths
- Consistence with previous studies
- The Norwegian strategy
- Advantages of using two models
- Limitations
- Future considerations and conclusion

- References: There is unnecessary duplication of the full bibliography, hence references in the main article only mentioned in the supplementary material. The main bibliography of the article should be reduced to the items cited.

- **We have updated the bibliography in the new pdf files.**
